# Functional models in genome-wide selection

**DOI:** 10.1371/journal.pone.0222699

**Published:** 2019-10-23

**Authors:** Ernandes Guedes Moura, Andrezza Kellen Alves Pamplona, Marcio Balestre

**Affiliations:** 1 Federal Institute of Maranhão - Campus São João dos Patos, São João dos Patos, Maranhão, Brasil; 2 Federal Institute of the Triângulo Mineiro – Campus Uberaba, Uberaba, Minas Gerais, Brasil; 3 Department of Statistics - Federal University of Lavras, Lavras, Minas Gerais, Brazil; National Center for Biotechnology Information, UNITED STATES

## Abstract

The development of sequencing technologies has enabled the discovery of markers that are abundantly distributed over the whole genome. Knowledge about the marker locations in reference genomes provides further insights in the search for causal regions and the prediction of genomic values. The present study proposes a Bayesian functional approach for incorporating the marker locations into genomic analysis using stochastic methods to search causal regions and predict genotypic values. For this, three scenarios were analyzed: F_2_ population with 300 individuals and three different heritability levels (0.2, 0.5, and 0.8), along with 12,150 SNP markers that were distributed through ten linkage groups; F_∞_ populations with 320 individuals and three different heritability levels (0.2, 0.5, and 0.8), along with 10,020 SNP markers that were distributed through ten linkage groups; and data related to *Eucalyptus spp*. to measure the model performance in a real LD setting, with 611 individuals whose phenotypes were simulated from QTLs distributed through a panel of 36,812 SNPs with known positions. The performance of the proposed method was compared with those of other genome selection models, namely, RR-BLUP, Bayes B and Bayesian Lasso. The Bayesian functional model presented higher or similar predictive ability when compared with those classical regressions methods in simulated and real scenarios on different LD structures. In general, the Bayesian functional model also achieved higher computational efficiency, using 12 SNPs per MCMC round. The model was efficient in the identification of causal regions and showed high flexibility of analysis, as it is easily adaptable to any genomic selection model.

## Introduction

One of the major interests of breeders is to develop high-performance cultivars for target traits without exhausting genetic variability [1]. Thus, major effort has been expended toward understanding the genetic mechanisms that are responsible for such traits [2,3] However, this task can be very difficult, since most of these quantitative traits can be controlled by several genes with small effects and a few with large ones [2,3]. In addition, several other genetic effects may be neglected, thereby increasing the so-called missing heritability [4,5].

The use of a high-density panel of markers that are associated with phenotypic information has enabled breeders/geneticists to represent part of the genetic architecture using a genome-wide framework. While the use of molecular markers has enabled new insights to be obtained about the genetic architecture of traits, the amount of available genomic information has increased significantly. Therefore, multiple statistical problems have emerged, e.g., multicollinearity, overfitting and genetic pitfalls such as false linkage disequilibrium due to the high model dimensionality, which arises from saturated panels and the limited availability of phenotypic information. Under this scenario, it is necessary to establish statistical approaches that relax these constraints, which are related to frequentist linear models. In this scenario, penalized estimators were proposed for genomic studies, such as parametric models that are based on mixed models (Ridge Regression Best Linear Unbiased Prediction—RR-BLUP, Genomic Best Linear Unbiased Prediction—GBLUP), semi-parametric models (kernel regressions), learning methods and Bayesian regressions (see [6]). The main advantage of such models has been widely discussed in Tempelman [6], Gianola et al. [7] and Gianola [8].

Genome-wide selection (GWS) was initially proposed by Meuwissen et al. [7]. These authors proposed the use of penalized regressions that are based on marker information and phenotypic data, with the aim of overcoming the over-parameterization problem. Given the shrinkage effect that is produced by Meuwissen’s models on the marker effects, the significance test on causal markers was neglected, while the prediction of genotypic values (GV) and the selection of the best individuals assumed a primary role. The predictions of GVs were obtained using a genotypic aggregate that was derived from a linear combination of the marker effect and its current state in an individual.

Based on this strategy, Bayesian genomic regression models and mixed model approaches have been proposed for dealing with a large Single Nucleotide Polymorphism (SNP) panel and limited phenotypic information. The main difference among the Bayesian methods is related to prior assumptions, which were named the Bayesian alphabet by Gianola et al. [8]. Furthermore, adapted models that are used in Quantitative Trati Loci (QTL) analysis, such as the shrinkage model of Xu [9] and stochastic search variable selection (SSVS), which was adapted by Yi et al. [10], have been proposed in the GWS context. However, as highlighted by Gianola et al. [8] and Gianola [8,11], many of these Bayesian models are highly influenced by assumptions about the hyperparameters of priors. Therefore, the information that is contained in the data may have little influence on the estimation of variance components and the relative entropy between the prior and posterior distributions is minimal. Tempelman [6] notes that due to this feature, the variance components in genomic regression models can be described as augmented data, rather than a measure of precision. Thus, these models would be better described as predictive machinery than genetic models.

To approximate the regression models for the genetic architecture, Hu et al. [12] developed the so-called continuous genome model, which is based on functional or pseudo-functional models. The genome is divided into lags of high linkage disequilibrium (which are called bins) by reformulating a type of haplotype model. By dividing the genome into bins, the haplotype (bin) effects are analyzed rather than individual marker effects, thereby reducing the dimension of the model and simplifying the Monte Carlo Markov Chain (MCMC) algorithms and Bayesian models.

The approach of Hu et al. [12] is founded on the idea that gene expression in the genome follows a spatial series whose function is unknown. Thus, gene expression signals are functions of the genome position on the chromosome. However, rather than searching for the functional description, Hu et al. [12] used bin marker clustering as a measure of information. The bin analysis that was proposed by these authors showed significant improvement in the predictive accuracy of GV when compared to competing methods such as eBayes, GBLUP, Bayes B1, Bayes B2 and Lasso in an oligogenic scenario [12].

The strategy of dividing the genome into bins has been successfully used in QTL mapping [13–16]. Genome intervals that are defined by the high linkage disequilibrium (LD) blocks were called natural bins by Xu [9]. A smoothing spline technique was considered by Beissinger et al. [17] for identifying threshold regions while considering inbreeding from SNP. However, Xu [9] stated that although natural bins have been shown to be effective in genomic prediction, the results may not be directly applied in some specific situations due to the sample size or the number of markers.

To overcome this problem, Xu [9] established the concept of artificial bins, in which breakpoints are allowed within natural bins or LD blocks and the number of artificial bins is determined in advance (fixed) by the investigator. However, the division of the genome into artificial bins was not clearly described and once these artificial bins have been established, the average SNP state (0, 1 or 2) is estimated. Moreover, the assumption of uniform LD at bins is very strong and important information can be neglected. Thus, searching for models in which the breakpoints are established directly from the data has become an attractive line of research.

An alternative to the method used by Xu [9] is to assume that candidate markers are random discrete variables that are distributed uniformly through the genome whose genomic signal function is unknown. In this case, stochastic methods such as random walks or Metropolis-Hastings algorithms may be used to integrate numerically the unknown function. Under high LD, the bins or knots will present no preferential SNP and the functional signal will remain constant in the genomic window.

A similar approach was widely used in QTL mapping, in which the spatial genomic series was delimited by flanking markers at specific positions in the genome [18]. Thus, the extension of this technique to genomic selection models can be easily adopted, thereby reducing the model size and the multicollinearity effects.

The present study proposes an alternative approach to dealing with continuous genome methods using functional models in the genomic selection framework and compares it with traditional methods. Additionally, we seek to identify possible breakpoint regions or natural bins using the signal function that is captured by genomic scanning.

## Material and methods

### Material

#### Simulated data

Some LD blocks configurations were simulated in this study in order to evaluate the bin performance in different mating scenarios. The first scenario, 12,150 SNP markers were uniformly distributed thought ten chromosomes with an average distance between them of 0.001 cM in the genome, totaling genome length of 1,200cM (120 cM per chromosome). The Haldane function was used as mapping function and random walk as meiosis method to build a SNP panel of 300 individuals in a F2 population using the QGenes software [19]. The second scenario consisted of an F_∞_ population with the same simulation settings of the first one, resulting in a total de 10,020 SNP markers in 320 individuals.

A total of 12 markers were assumed to be QTLs in both scenarios and their effects were sampled from a normal distribution with zero mean and a standard deviation of one. The individual genotypic values were constructed by the linear combination of QTL effects with the QTL genotype under three heritability scenarios: 0.2, 0.5 and 0.8. The Gaussian residuals were sampled according to requested heritability. Multiple artificial bins were adopted in these heritability scenarios: Bin 0.01, Bin 0.005 and Bin 0.001. The sizes of the bins are, respectively, 0.82%, 1.64%, and 8.23% of the number of markers in the F_2_ population (corresponding to 121, 60, and 12 bins in the genome) and 0.99%, 1.99%, and 9.98% of the number of markers in the F_∞_ population (corresponding to 100, 50, and 10 bins in the genome.

In the third scenario, a natural LD structure of *Eucalyptus* spp was used to simulate the phenotypes related to 611 individuals. The SNP panel was 36,812 markers distributed through ten chromosomes with known SNP position (kb). The phenotypes also were built through 12 simulated QTLs whose effects were sampled from a standard Gaussian distribution and the position sampled from a uniform distribution. The Gaussian residues were sampled from a Gaussian with variances related to three simulated heritability; 0.2, 0.5 and 0.8. The use of real LD structure is important to evaluate the model performance in low LD blocks scenario. In addition, true phenotypic data was used for these 611 plants. More details about the *Eucalyptus* background is given above.

In the last scenario, human genome panel contained about 9,307 SNPs from the HapMap project distributed across 22 chromosomes was used. Information about two different populations is available in https://cran.r-project.org/web/packages/SNPassoc/SNPassoc.pdf: European population (CEU) and Yoruba (YRI) [20]. The genomic information (names of SNPs, chromosomes and genetic position) is also available in a data frame called HapMap.SNPs.pos. The SNP panel was corrected by MAF and the missed values inputted by A.Mat function available in GBLUP library and a final panel 7,574 SNPs and 120 individuals were used for performing GS. The simulation settings were the same those used in plant simulation.

#### Real data

For illustrate the bin model on real phenotypic data, a Eucalyptus spp. population that was composed of 611 individuals was used. This population is traced back to crosses that involved E. grandis, E. urophylla, E. globulus and E. camaldulensis. The true phenotypic data used here was the circumference at breast height (CBH) measure was collected at 24 months of age (two years). The SNP panel used was the same used for simulated data but now using real phenotypic information. The DNA from all 611 plants of this population was extracted at two years of age and genotyping was performed with 36,812 SNPs. For the real Eucalyptus data, 100 bins were spread across the genome; each bin was formed by 368 SNPs, except the last bin, which had 380 SNPs.

### Methods

#### Functional model

**Fig 1:**
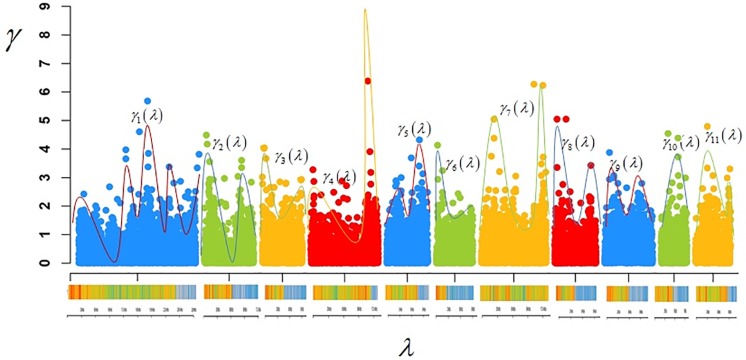
The functional continuous profile related to a hypothetical genome. Here *γ* is the additive effect, *λ* the SNP genome position and *γ*(*λ*) the functional relationship among the SNP position with the additive effect. The classical genome selection models search to find the marker effect *γ* related to SNP (colored dot). Functional models search to find the function *γ*(*λ*) relating the markers position *λ* to its effects *γ*.

The functional genomic model is based on the premise that the functional signal of a gene through the genome can be described by a one-dimensional function (Fig 1). In this figure, each dot representing a candidate region can be smoothed by a functional relationship between the genome position *λ* and the candidate region presenting signal *γ*.

As showed by Xu [9] the integration of *γ*(*λ*) (Fig 1) returns the predictive additive value ($\hat{VGA}$) of the *i*-th individual $\hat{VGA}=\int z{\left(\lambda \right)}_{}\hat{\gamma }(\lambda )d\lambda$ while in classical genomic selection models the integrant is replaced by the sum of the linear combination among SNP genotypes and their additive effects $\hat{VGA_{i}}=\sum_{j=1}^{p}z_{ij}{\hat{\gamma }}_{j}$ where *p* is the size of SNP panel.

Thus, based on the Fig 1, if we have C chromosomes in the genome and C functions, the genomic functional model can be rewritten in a piecewise functional form:

$$y_{i}=\mu +\sum_{j=1}^{C}\int_{0}^{L}Z_{ij}(\lambda )\gamma (\lambda )d\lambda +\varepsilon_{i},\;\forall i=1,\ldots ,n$$ (1)

where the summation describes the discontinuity of the function along the chromosomes and *L*_*j*_ is the size of chromosome *j*. Under this model, the continuous genotypic state **Z**(*λ*) is not entirely known, except at the available markers. Note that what **Z** does is consider how much each point *γ* will contribute to the integral in (1) according to the genomic status (2,1 and 0) of the *i*-th individual for a specific position *λ* and, consequently, to predict *y*_*i*_. That is, which loci influence more on the response variable. More detail about the model justification is given in supplemental material (S1 Text).

Since the function *γ*(*λ*) is unknown, the integral in (1) is not explicit and numerical integration is necessary. There are various algorithms for performing numerical integration; in this study, the chromosome division strategy at bins was adopted. Within each bin, the unknown continuous function *f*(*λ*) = *γ* can be estimated empirically using the pair (*λ*_*j*_; *γ*_*j*_), i.e. for each genomic position must be a response in *γ* and vice-versa. This relationship can be used when *λ* is known for a specific marker. In this case, *γ*(*λ*) ≅ (*λ*_*j*_; *γ*_*j*_) or for each discretized position (*λ*_*j*_) there is a functional response *γ*_*j*_. Given that the effect of the pair (*λ*_*j*_; *γ*_*j*_) is unknown, it can only be obtained using the observed position *λ* and the scalar phenotype (*y*). Then, (*λ*_*j*_; *γ*_*j*_) can be considered a random variable whose posterior distribution can be obtained by $p(\gamma |\lambda ,y)=\frac{p(y)p(\gamma |y)p(\lambda |\gamma ,y)}{p(y)p(\lambda )}=\frac{p(\gamma |y)p(\lambda |\gamma ,y)}{p(\lambda )}\propto p(\gamma |y)p(\lambda |\gamma ,y)$. Here, the proportional means that the marginal distribution *p*(*λ*) is a uniform (constant) distribution while *p*(*λ*)|*γ*, *y*) depend on additive effect and the phenotypic value respectively. If the model parameters do not inform about the SNP position then: *p*(*λ*)|*γ*, *y*) = *p*(*λ*) and the additive effect will depend only of the phenotypic value.

The model above assumes conditional independency between *λ* and y since the phenotypic value y informs about *λ* through *γ*. The pair (*λ*_*j*_; *γ*_*j*_) can be estimated using the maximum posterior of *p*(*γ*|*λ*, *y*) as ${\hat{\gamma }}_{j}|\lambda_{j},y≙\underset{\gamma \in ℜ}{\text{arg}.\text{max}}\left[p(\gamma |y)\right]p(\lambda |\gamma ,y)$; however, *p*(*λ*|*γ*, *y*) ≔ {*λ*|*λ* ∊ *S* ≡ [*λ*_*k*(min)_, *λ*_*k*(max)_]} is unknown on the *k*-th bin, but it can be integrated numerically through MCMC using the Metropolis-Hastings method.

#### Prior distributions

Using the relationship described above, namely, *f*(*λ*) = *γ* ∼ *γ*(*λ*), then *γ* is a signal/functional parameter to be estimated. In the Bayesian context, the observable variables are the phenotypic values (***y***), the marker genotypes (**Z**) and the positions of markers *λ* in the reference genome. The non-observable variables are the regression coefficients **μ** and *γ* the variances $\sigma_{e}^{2}$ and $\sigma_{\gamma }^{2}$. The Jeffrey’s priors were assumed for the residual variance and the general means, which are given by:

$$p\left(\mu \right)\propto 1,\;p\left(\sigma_{e}^{2}\right)\propto \frac{1}{\sigma_{e}^{2}}$$ (2)

The prior distributions for the marker effect *γ* and its variance were assumed to be:

$$p\left(\gamma_{m}\right)\propto N\left(0,\sigma_{\gamma_{m}}^{2}\right),\;p\left(\sigma_{\gamma_{m}}^{2}\right)\propto \chi_{esc}^{-2}\left(\upsilon ,S^{2}\right)$$ (3)

where *γ*_*m*_ is the *m*-th marker effect on the position *λ*_*m*_, and *υ* = 1 and $S^{2}=\frac{\sigma_{y}^{2}\cdot 0.01}{M^{2}}$ are respectively the degree of freedom and the scale parameter for the marker variances, and M is the number of markers.

The marker positions satisfy a biunivocal relation with the marker status (*λ*_1_ = *M*_1_, *λ*_2_ = *M*_2_, …, *λ*_*m*_ = *M*_*m*_); therefore, there is an equivalence between the sampled marker genotype **Z**(*λ*) and its position *λ*.

To simplify the notation, by taking **γ** = {*γ*_*m*_}, $\sigma_{\gamma }^{2}=\left\{\;\sigma_{\gamma_{m}}^{2}\right\}$ and **λ** = {*γ*_*m*_}, the joint prior distribution for unobservable variables is given by:

$$p\left(\mu ,\sigma_{e}^{2},\gamma ,\sigma_{\gamma }^{2}\right)\propto p\left(\mu \right)p\left(\sigma_{e}^{2}\right)\prod_{m=1}^{M}p\left(\gamma_{m}|\lambda \right)p\left(\sigma_{\gamma }^{2}\right)$$ (4)

where *M* is the total number of markers and positions in the marker panel.

#### Joint likelihood of phenotype and marker position

The joint likelihood for the phenotypic observation and marker position is expressed as:

$$p\left(y,\lambda |\mu ,\sigma_{e}^{2},\gamma ,\sigma_{\gamma }^{2}\right)=p\left(y|\mu ,\sigma_{e}^{2},\gamma ,\sigma_{\gamma }^{2}\right)p\left(\lambda |\mu ,\sigma_{e}^{2},\gamma ,\sigma_{\gamma }^{2},y\right)$$ (5)

The likelihood for the phenotypic data under the complete model is described by:

$$\begin{array}{l} p\left(y|\mu ,\sigma_{e}^{2},\gamma ,\sigma_{\gamma }^{2}\right)=\overset{n}{\underset{i=1}{\prod }}p\left({\text{y}}_{i}|\mu ,\sigma_{e}^{2},\gamma ,\sigma_{\gamma }^{2}\right)\\ \;\propto {\left(\sigma_{e}^{2}\right)}^{-n/2}exp\left\{-\frac{1}{2\sigma_{e}^{2}}\overset{n}{\underset{i=1}{\sum }}{\left(y_{i}-\mu -\overset{C}{\underset{j=1}{\sum }}\overset{L_{j}}{\underset{0}{\int }}Z_{ij}(\lambda )\gamma (\lambda )d\lambda \right)}^{2}\right\} \end{array}$$ (6)

which can be approximated by:

$$p\left(y|\mu ,\sigma_{e}^{2},\gamma ,\sigma_{\gamma }^{2}\right)=\propto {\left(\sigma_{e}^{2}\right)}^{-n/2}exp\left\{-\frac{1}{2\sigma_{e}^{2}}\sum_{i=1}^{n}{\left(y_{i}-\mu -\;Z_{i}P_{\lambda }\hat{\gamma }\right)}^{2}\right\}$$ (7)

where *P*_*λ*_ is the weight function, which will be described later, and **y** = {*y*_1_, *y*_2_,…,*y*_*n*_} is the vector of phenotypic observations.

Through Bayes’ theorem, the posterior distribution can be described by:

$$p\left(\mu ,\sigma_{e}^{2},\gamma ,\sigma_{\gamma }^{2}|y,\lambda \right)\propto p\left(y|\mu ,\sigma_{e}^{2},\gamma \right)p\left(\lambda |\mu ,\sigma_{e}^{2},\gamma ,\sigma_{\gamma }^{2},y\right)p\left(\mu ,\sigma_{e}^{2},\gamma ,\sigma_{\gamma }^{2}\right)$$ (8)

where $p\left(\lambda |\mu ,\sigma_{e}^{2},\gamma ,\sigma_{\gamma }^{2},y\right)$ is the unknown distribution conditioned on all model parameters. In this case, the objective is to find $p\left(\lambda |\mu ,\sigma_{e}^{2},\gamma ,\sigma_{\gamma }^{2},y\right)$; this function can be integrated numerically with the Metropolis-Hastings algorithm, given the conditioning elements of the model and using a uniform distribution [*λ*_(min)_, *λ*_(max)_] to wrap $p\left(\lambda |\mu ,\sigma_{e}^{2},\gamma ,\sigma_{\gamma }^{2},y\right)$. Thus, for a bin range [*λ*_(min)_, *λ*_(max)_], each *λ* can be considered a discrete element and $\underset{N\to \infty }{\text{lim}}\left[p\left(\lambda |\mu ,\sigma_{e}^{2},\gamma ,\sigma_{\gamma }^{2},y\right)≙f(\lambda |\mu ,\sigma_{e}^{2},\gamma ,\sigma_{\gamma }^{2},y)\right]$, i.e., the continuous probability function can be approximated by the frequency distribution given the rate of visit to *λ* along the *N* runs of the MCMC algorithm. Thus, $f(\lambda |\mu ,\sigma_{e}^{2},\gamma ,\sigma_{\gamma }^{2},y)=P_{\lambda }$ and the previously described relationship can be approximated by $\hat{\gamma }|\lambda ≙\underset{\gamma \in ℜ}{\text{arg}.\text{max}}\left[p(\gamma |y)\right]p(\lambda |\gamma ,y)≐\underset{\gamma \in ℜ}{\text{arg}.\text{max}}\left[p(\gamma |y)\right]P_{\lambda }$.

#### Markov chain Monte Carlo for functional genomic models

The MCMC by Gibbs sampling was used for the model parameters and the Metropolis-Hastings method was used for the numerical integration of $p\left(\lambda |\mu ,\sigma_{e}^{2},\gamma ,\sigma_{\gamma }^{2},y\right)$. To perform the numerical integration, the genome was divided into bins and a position *λ*_*j*_ was drawn from each bin. Therefore, the model dimension for each MCMC run was restricted to the number of bins *k*. The steps are described as follows:

1. Initialization: The parameters μ and $\sigma_{e}^{2}$ are initialized using the mean and variance of the phenotypic data, respectively; the vector *γ* is initialized to a value zero and dimension *K*, where *K* is the number of bins in the current model. The matrix that is related to genotypic state *Z*_*λ*_ (*n* × *K*) was sampled from the complete matrix **Z** (*n* x *M*), where *M* ≥ *K* and the index *λ*_*k*_ corresponds to the initial marker position that is sampled in the *k-*th bin. In this way, $Z_{\lambda_{k}}$ was initially sampled on the basis of the median position *λ*_*k*_ in the *k-*th bin. The effect variances of each marker $\sigma_{\gamma }^{2}$ were initially assumed to be non-null (0.5). The initial vector can be represented by:
  $$I^{\left(0\right)}=\left[\mu^{\left(0\right)},\gamma_{1}{}^{\left(0\right)},\ldots ,\gamma_{K}{}^{\left(0\right)},\sigma_{e}^{2}{}^{{}^{\left(0\right)}},\sigma_{\gamma_{1}}^{2^{\left(0\right)}},\ldots ,\sigma_{\gamma_{K}}^{2^{\left(0\right)}},Z_{\lambda_{K}}\right]$$ (9)
2. In this step, *μ* is updated from the following Gaussian conditional distribution:
  $$\mu |\ldots ∼N\left[\sum_{i=1}^{n}\left(y_{i}-\sum_{k=1}^{K}Z_{\lambda_{k}}\gamma_{\lambda_{k}}\right)/n,\;\frac{\sigma_{e}^{2\;}}{n}\right]$$ (10)
  Note that the index of summation is K, not *M*. This is justified by the Bernoulli process that is observed in stochastic searches during the MCMC, i.e., since *λ*_*k*_ is sampled at the *t*-th iteration, $p_{\lambda_{k}^{t}}=1$ and $p_{\lambda_{\neg k}^{t}}=0$ for a given bin. Thus, at the *t*-th iteration, $Z_{\lambda^{t}}P_{\lambda^{t}}{\hat{\gamma }}_{\lambda^{t}}=\sum_{k=1}^{K}Z_{\lambda_{k}}\gamma_{\lambda_{k}}$, where $P_{\lambda^{t}}$ is a diagonal binary matrix (*M* x *M*) that indicates which marker is being evaluated within the *k*-th bin in the *t*-th iteration. This process makes the bin model faster.
3. The marker effects ($\gamma_{\lambda_{k}^{t}}$) are sampled from a Gaussian posterior distribution given the actual position *λ*_*k*_ using the following distribution:
  $$\gamma_{\lambda_{k}^{t}}|\ldots N\left({\left(\sum_{i=1}^{n}Z_{\lambda_{k_{\left(i\right)}}^{t}}^{2}+\frac{\sigma_{e}^{2\;}}{\sigma_{\gamma_{\lambda_{k}^{t}}}^{2\;}}\right)}^{-1}\sum_{i=1}^{n}Z_{\lambda_{k_{\left(i\right)}}^{t}}\left(y_{i}-\mu -\sum_{k´=1}^{K-1}Z_{\lambda_{k{´}_{\left(i\right)}}^{t}}\gamma_{\lambda_{k´}^{t}}\right),{\left(\sum_{i=1}^{n}Z_{\lambda_{k_{\left(i\right)}}^{t}}^{2}+\frac{\sigma_{e}^{2\;}}{\sigma_{\gamma_{\lambda_{k}^{t}}}^{2\;}}\right)}^{-1}\sigma_{e}^{2}\right)$$ (11)
  which is obtained from:
  $$p\left(\gamma_{\lambda_{k}^{t}}|\ldots \right)\propto \text{exp}\left\{-\frac{1}{2\sigma_{e}^{2}}\sum_{i=1}^{n}{\left(y_{i}-\mu -\sum_{k=1}^{K}Z_{\lambda_{k}}\gamma_{\lambda_{k}}\right)}^{2}\right\}\text{exp}\left\{-\frac{1}{2\sigma_{\gamma_{{}_{\lambda_{k}}}}^{2}}\sum_{k=1}^{K}\gamma_{{}_{\lambda_{k}}}^{2}\right\}$$ (12)
4. As mentioned above, $p\left(\lambda |\mu ,\sigma_{e}^{2},\gamma ,\sigma_{\gamma }^{2},y\right)$ is unknown and the Metropolis-Hastings algorithm [21,22] can be used since it does not require a closed form of the conditional distribution. In this sense, from an auxiliary distribution, it is possible to sample *λ* conditional to all model parameters using *α* as an acceptability criterion.
  In this case, a uniform distribution was used to wrap the *λ*_*k*_ distribution, where *λ*_*k*_ was sampled within the bin that is delimited by *max*(*LI*_*k*_, *λ*_*k*_ − *c*) and *min*(*LS*_*k*_, *λ*_*k*_ + *c*), where *c* is a constant that defines the tuning in the *k*-th bin, which is usually fixed at 1% of the bin range. This uniform distribution is denoted by $u\left(\lambda_{k}^{NEW},\lambda_{k}^{\left(t\right)}\right)$ for the *k-*th bin and the new position $\lambda_{k}^{NEW}$ is accepted in the *t-*th iteration with probability *min*(1, *α*_*k*_). Thus, if *α*_*k*_ is accepted, a new position is established, which is denoted by $\lambda_{k}^{NEW}$, and the marker status $Z_{\lambda_{k}^{NEW}}$ is sampled from the complete panel. The decision rule for accepting the new marker position within the bin is given by:
  $$\alpha_{k}=\frac{p\left(\lambda^{NEW}|\mu ,\sigma_{e}^{2},\gamma ,\sigma_{\gamma }^{2},y\right)u\left(\lambda^{NEW},\lambda_{k}^{\left(t\right)}\right)}{p\left(\lambda_{k}^{\left(t\right)}|\mu ,\sigma_{e}^{2},\gamma ,\sigma_{\gamma }^{2},y\right)u\left(\lambda_{k}^{\left(t\right)},\lambda^{NEW}\right)}$$ (13)
  where
  $$p\left(\lambda^{NEW}|\mu ,\sigma_{e}^{2},\gamma ,\sigma_{\gamma }^{2},y\right)\propto \text{exp}\left\{-\frac{1}{2\sigma_{e}^{2}}\sum_{i=1}^{n}{\left(y_{i}-\mu -\sum_{k=1}^{K-1}Z_{\lambda_{k}}\gamma_{\lambda_{k}}-Z_{\lambda^{NEW}}\gamma_{\lambda^{NEW}}\right)}^{2}\right\}$$ (14)
  and
  $$p\left(\lambda_{k}^{\left(t\right)}|\mu ,\sigma_{e}^{2},\gamma ,\sigma_{\gamma }^{2},y\right)\propto \text{exp}\left\{-\frac{1}{2\sigma_{e}^{2}}\sum_{i=1}^{n}{\left(y_{i}-\mu -\sum_{k=1}^{K}Z_{\lambda_{k}}\gamma_{\lambda_{k}}\right)}^{2}\right\}$$ (15)
  The Hastings correction is given by:
  $$\begin{array}{ll} u\left(\lambda_{k}^{\left(t\right)},\lambda^{NEW}\right) & =\{\begin{array}{ll} \frac{1}{2c}, & \text{if}\;\lambda_{k}^{\left(t\right)}+c\leq LS_{k}\;\text{and}\;\lambda_{k}^{\left(t\right)}-c\geq LI_{k}\\ \frac{1}{c+\lambda_{k}^{\left(t\right)}-LI_{k}}, & \text{if}\;\lambda_{k}^{\left(t\right)}+c<LS_{k}\;\text{and}\;\lambda_{k}^{\left(t\right)}-c<LI_{k}\\ \frac{1}{c+LS_{k}-\lambda_{k}^{\left(t\right)}}, & \text{if}\;\lambda_{k}^{\left(t\right)}+c>LS_{k}\;\text{and}\;\lambda_{k}^{\left(t\right)}-c>LI_{k} \end{array} \end{array}$$
  $$\begin{array}{ll} u\left(\lambda^{NEW},\lambda_{j}^{\left(t\right)}\right)= & \{\begin{array}{ll} \frac{1}{2c}, & \text{se}\;\lambda^{NEW}+c\leq LS_{j}\;\text{e}\;\lambda^{NEW}-c\geq LI_{j}\\ \frac{1}{c+\lambda^{NEW}-LI_{j}}, & \text{se}\;\lambda^{NEW}+c<LS_{j}\;\text{e}\;\lambda^{NEW}-c<LI_{j}\\ \frac{1}{c+LS_{j}-\lambda^{NEW}}, & \;\text{se}\;\lambda^{NEW}+c>LS_{j}\;\text{e}\;\lambda^{NEW}-c>LI_{j} \end{array} \end{array}$$ (16)
5. The conditional for the residual variance after accepting the *m-*th position in the genome is an inverted chi-squared distribution:
  $$\sigma_{e}^{2}|\ldots ∼\chi_{esc}^{-2}\left(n,{\sum_{i=1}^{n}\left(y_{i}-\mu -\sum_{k=1}^{K}Z_{\lambda_{k}}\gamma_{\lambda_{k}}\right)}^{2}\right)$$ (17)
6. Finally, the marker-specific variance $\sigma_{\gamma_{{}_{\lambda }}}^{2}$ is sampled using an inverted chi-squared distribution:
  $$\sigma_{\gamma_{\lambda }}^{2}|\ldots ∼\chi_{esc}^{-2}\left(\upsilon +1,\;\gamma_{\lambda }^{2}+S^{2}\right)$$ (18)

Upon chain convergence, the relationship $f(\lambda |\mu ,\sigma_{e}^{2},\gamma ,\sigma_{\gamma }^{2},y)=P_{\lambda }$ was adopted as the frequency distribution, which is related to the number of beats at position *λ* within a specific bin. However, the integral $\int_{0}^{L}Z(\lambda )\gamma (\lambda )d\lambda$ was used to recompose the genomic genetic value and *γ*(*λ*) remains unknown. Hu et al. [12] used the average effect of bins as the expectation of the uniform distribution $p\left(\lambda |\mu ,\sigma_{e}^{2},\gamma ,\sigma_{\gamma }^{2},y\right)$. In the present study, the assumption of the uniform distribution is relaxed since for each *λ*, it is attributed number of beats, thereby making it possible to obtain distributions of several shapes and to approximate $\int_{0}^{L}Z(\lambda )\gamma (\lambda )d\lambda$ by $ZP_{\lambda }\hat{\gamma }$, as was already demonstrated above. A faster approach is to use the mean of the chains that are related to marker effects, thereby avoiding designing an additional weight function. It can be obtained with MCMC on the *t*-th iteration by assigning the null effect ($\hat{\gamma }=0$) to all markers whose positions are not visited. Taking *ψ* as the MCMC chain mean after convergence, we have $\psi =\frac{\sum_{l=1}^{N}{\hat{\gamma }}_{l}}{N}=\frac{\tau \sum_{l=1}^{\tau }{\hat{\gamma }}_{l}+\tau (N-\tau )\times 0}{\tau N}=\frac{\tau }{N}\bar{\gamma }={\hat{P}}_{\lambda }\bar{\gamma }$, where *N* is the total size of the chain, τ is the number beats at the *m*-th marker position during the MCMC process and ${\hat{P}}_{\lambda }=\frac{\tau }{N}$. Under a posterior Gaussian distribution, we have $\psi ={\hat{P}}_{\lambda }\bar{\gamma }=P_{\lambda }\underset{\gamma \in ℜ}{\text{arg}.\text{max}}\left[p(\gamma |y)\right]$. Thus, the prediction of the final genomic genetic value is given by $Z\psi =\hat{g}$.

#### Implementation of the analysis

The algorithm for the Gibbs sampler and Metropolis-Hastings was implemented using R software [23]. The chain size of 5000 samples was considered after burning the first 1000 iterations and thinning two every other samples. All data and R library BFBM 1.0 are available in the supplemental files.

#### Model predictive ability

To compare the functional model, classical genome selection models were used, such as the Bayes B and Bayesian Lasso models, with the BGLR function of the BGLR package [24], functional model, with the binmod function of the PAS package [25] and the RR-BLUP model with the mixed.solve function [26] of the RR-BLUP package; All libraries are available in R software CRAN [24]).

To evaluate the predictive ability relative to true simulated values, the mean squared error (MSE) was used, which is defined by:

$$\text{EQM}=\frac{1}{n}\sum_{i=1}^{n}{\left(g_{i}-{\hat{g}}_{i}\right)}^{2}=\frac{1}{n}\sum_{i=1}^{n}{\left(y_{i}-\hat{\mu }-Z\psi \right)}^{2}$$ (19)

In addition to MSE, the coefficient of determination R² was estimated between predicted genetic value ${\hat{g}}_{i}$ and true value *g* through linear regression.

A cross-validation procedure was performed to evaluate the model’s ability to predict untested genotypes. For this, a 5-fold procedure was applied on all heritability scenarios and R² between the predicted and observed values was estimated. For real data, 6-fold cross-validation was performed.

## Results

### Simulated data analysis

**Fig 2:**
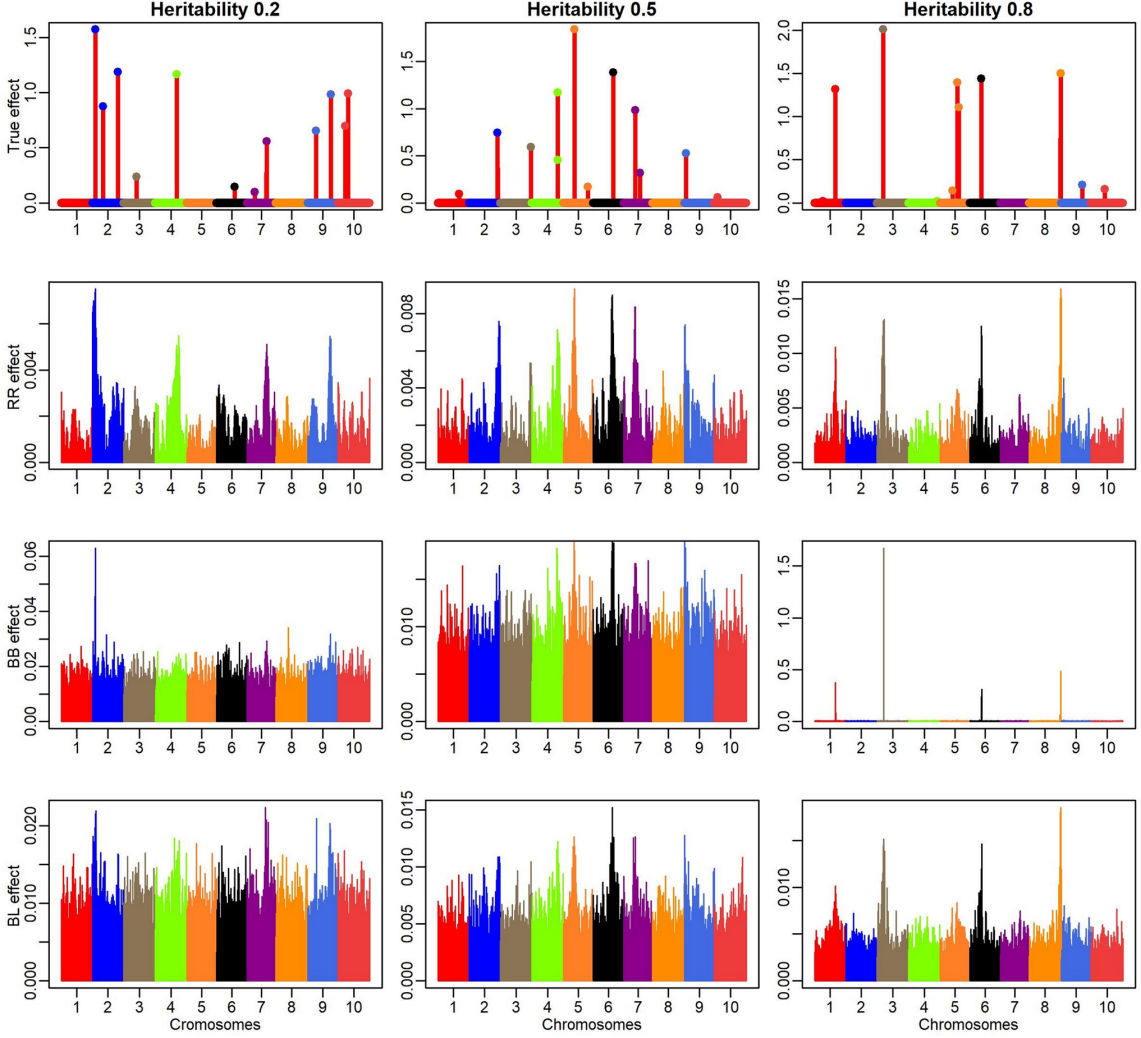
Absolute simulated actual effects of QTL along the genome and absolute estimates from methods rr-BLUP (RR), Bayes B (BB) and Bayesian Lasso (BL). Colored dots represent the 12 true QTL, which are distributed across 12,150 SNP over 10 linkage groups. There is a scale difference.

Fig 2 shows the absolute effects of the simulated QTL and the absolute effects that were obtained by RR-BLUP (RR), Bayes B (BB) and Bayesian Lasso (BL) for different levels of heritability. It was observed that the QTL that presented large effects were mapped by the three methods and the genomic distribution of the effects followed a similar pattern. However, in general, it presented a strong shrinkage effect, thereby underestimating the true values, with the exception of BB with the heritability level of 0.8. In this scenario, Bayes B has outperformed RR-BLUP and Bayesian Lasso, since its predicted values for the four QTL were similar to the simulated values. For the other heritability levels, the BB method performed similarly to the others. As expected, as the heritability increases, the resolution of methods in defining causal regions also increases producing a spike effect on the simulated QTL. However, even with high heritability, RR and BL showed downward bias due to the shrinkage effect.

**Fig 3:**
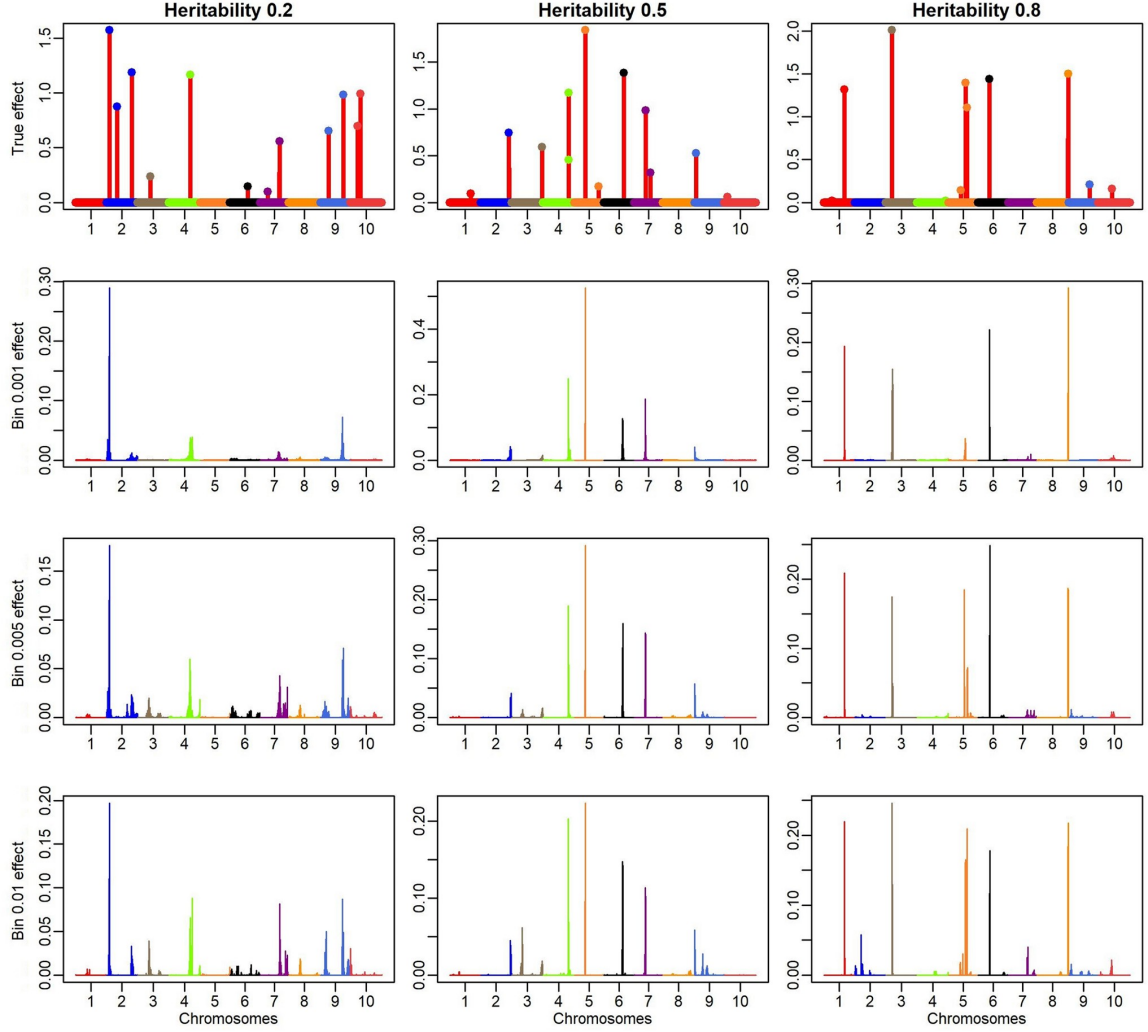
Absolute simulated actual effects of QTL along the genome and absolute estimates from the methods for Bin 0.001, Bin 0.005 and Bin 0.01. Colored dots represent the 12 true QTL, which are distributed across 12,150 SNP over 10 linkage groups. There is a scale difference.

Fig 3 shows the absolute effects of the simulated QTL and the absolute effects that were predicted by the methods for Bin 0.01, Bin 0.005 and Bin 0.001 for the three heritability levels. It is observed that most QTL with large simulated effects were mapped by all bin models, i.e., the effects that were predicted by the bin models showed similar patterns and the bin models generally obtained estimates that were closer to the true values, thereby presenting better genome resolution than traditional models (RR, BB and BL). The genomic profile at Bin 0.001 was similar to that obtained by BB for heritability level 0.8. However, it had the advantage of identifying an additional QTL on Chromosome 5, where three causal signals were simulated. Moreover, for lower heritability levels, the genome profile that was obtained by the Bin method remained constant. In summary, only on high heritability the Bayesian GS models (more specifically the Bayes B) was able to remove the LD noise on the SNP effect (high resolution mapping) indicating the power of bins model to remove this bias even in low heritability’s scenarios.

Another interesting result was the power of the Bin method in identifying QTL on the same chromosome. This result can be observed in bins 0.01 and 0.005. For Bin 0.01, the three QTL were well represented, including the effect magnitude. However, false-positive signs were found in chromosomes 2 and 7 in this bin configuration.

Table 1 presents the mean squared error (MSE) and the coefficient of determination (R²) between the true and predicted values using the considered methods under different scenarios of heritability.

**Table 1:** Mean Square Error (MSE) and coefficient of determination (R²) of the RR-BLUP, Bayes B, Bayesian Lasso, Bin 0.01, Bin 0.005 e Bin 0.001 models, for the three heritabilities considering three population structures.

Population	Models	Heritabilities					
		0.2		0.5		0.8	
		MSE	R^2^	MSE	R^2^	MSE	R^2^
F_2_	rr-BLUP	1.171	0.736	0.902	0.843	0.638	0.896
	Bayes B	1.587	0.676	0.882	0.846	0.543	0.927
	Bayesian Lasso	1.546	0.685	0.859	0.851	0.637	0.888
	Bin 0.01	1.187	0.815	0.884	0.871	0.400	0.963
	Bin 0.005	1.161	0.813	0.871	0.875	0.442	0.958
	Bin 0.001	0.993	0.838	0.747	0.903	0.398	0.961
	XU	0.683*	0.784	0.571**	0.882	0.420***	0.942
F_∞_	rr-BLUP	2.183	0.496	1.488	0.766	0.932	0.908
	Bayes B	2.121	0.524	1.286	0.825	0.668	0.952
	Lasso Bayesiano	2.183	0.496	1.461	0.774	0.923	0.910
	Bin 0.01	2.026	0.566	1.117	0.868	0.696	0.948
	Bin 0.005	1.946	0.600	1.022	0.889	0.830	0.927
	Bin 0.001	1.746	0.678	1.666	0.706	1.427	0.785
	XU	1.642*	0.447	0.857***	0.841	0.699***	0.921
*Eucalyptus*	rr-BLUP	2.219	0.586	1.487	0.784	0.475	0.910
	Bayes B	2.147	0.452	1.363	0.765	0.873	0.903
	Lasso Bayesiano	2.100	0.505	1.745	0.773	0.457	0.901
	Bin 0.01	2.021	0.558	0.995	0.785	0.385	0.915
	Bin 0.005	2.245	0.625	1.078	0.790	0.245	0.913
	Bin 0.001	2.075	0.641	0.925	0.813	0.285	0.927
	XU	1.386#	0.529	1.247^#^	0.636	0.514^##^	0.951
*Humans*	rr-BLUP	1.681	0.306	1.276	0.570	0.837	0.812
	Bayes B	2.184	0.274	1.189	0.589	0.623	0.846
	Lasso	2.176	0.273	1.011	0.582	0.521	0.775
	Bin 0.01	2.032	0.400	1.066	0.642	0.519	0.905
	Bin 0.005	2.273	0.368	1.044	0.644	0.510	0.904
	Bin 0.001	0.944	0.407	0.654	0.781	0.532	0.838
	XU	2.871*#	0.266	1.604^#*^	0.547	0.585^###^	0.844

* Best Xu model with 10 bins.

^#^ Best Xu model with 2 bins.

** Best Xu model with 90 bins.

^##^Best Xu model with 36812 bins.

*** Best Xu model with 190 bins.

*^#^ Best Xu model with 191 bins

^#^*Best Xu model with 370 bins

^###^ Best Xu model with 2741 bins

In the F_2_ population, it was observed that the regression models that were used in this study performed very differently according to the heritability scenario, i.e., RR-BLUP performed best for heritability level 0.2, Bayesian Lasso for heritability level 0.5, and Bayes B for heritability level 0.8. The best configuration of Xu et al. (2012) model performed better than Bayesian regression models, but it was less accurate than the bin model configurations. The bin model outperformed all models in terms of predictive ability and had the highest values of R^2^. However, the lowest values of MSE occurred for the Xu et al. (2012) model in the heritabilities 0.2 and 0.5, and when the heritability level was high, the lowest value of MSE occurred for the Bin 0.001 (1000 marker per bin). However, bin methods generally show high predictive ability of the true genomic genetic value.

In the F_∞_ population, it was observed that Bayes B was better among the Bayesian regression models and rr-BLUP in all heritabilities. The Xu et al. (2012) model outperformed the regression models only in the heritability 0.5, but in all scenarios it was overcome by the bin configurations. In this LD configuration, the bin model superiority in predicting the true values were slightly inferior to F_2_ populations. However, the lowest values of MSE occurred in the Xu et al. (2012) model in the heritabilities 0.2 and 0.5, and when the heritability level was high, the lowest value of MSE occurred for the Bayes B.

For humans dataset the Xu model presented the worst results requesting models where the number of bins ranged from 161 to the total panel of SNPs. The number of bin used our model was resilient to different LD structures presented good prediction ability independent from the population. In general the bins models performed very well in this LD structure independently of the bins number. Also, the bin model presented the best resolution in the genome scanning, presenting estimate of additive effect closer to simulated one (S10 and S11 Figs). This result has confirmed the model ability to deal problems related to biased estimates of the true additive values in which has been observed in shrinkage models.

**Fig 4:**
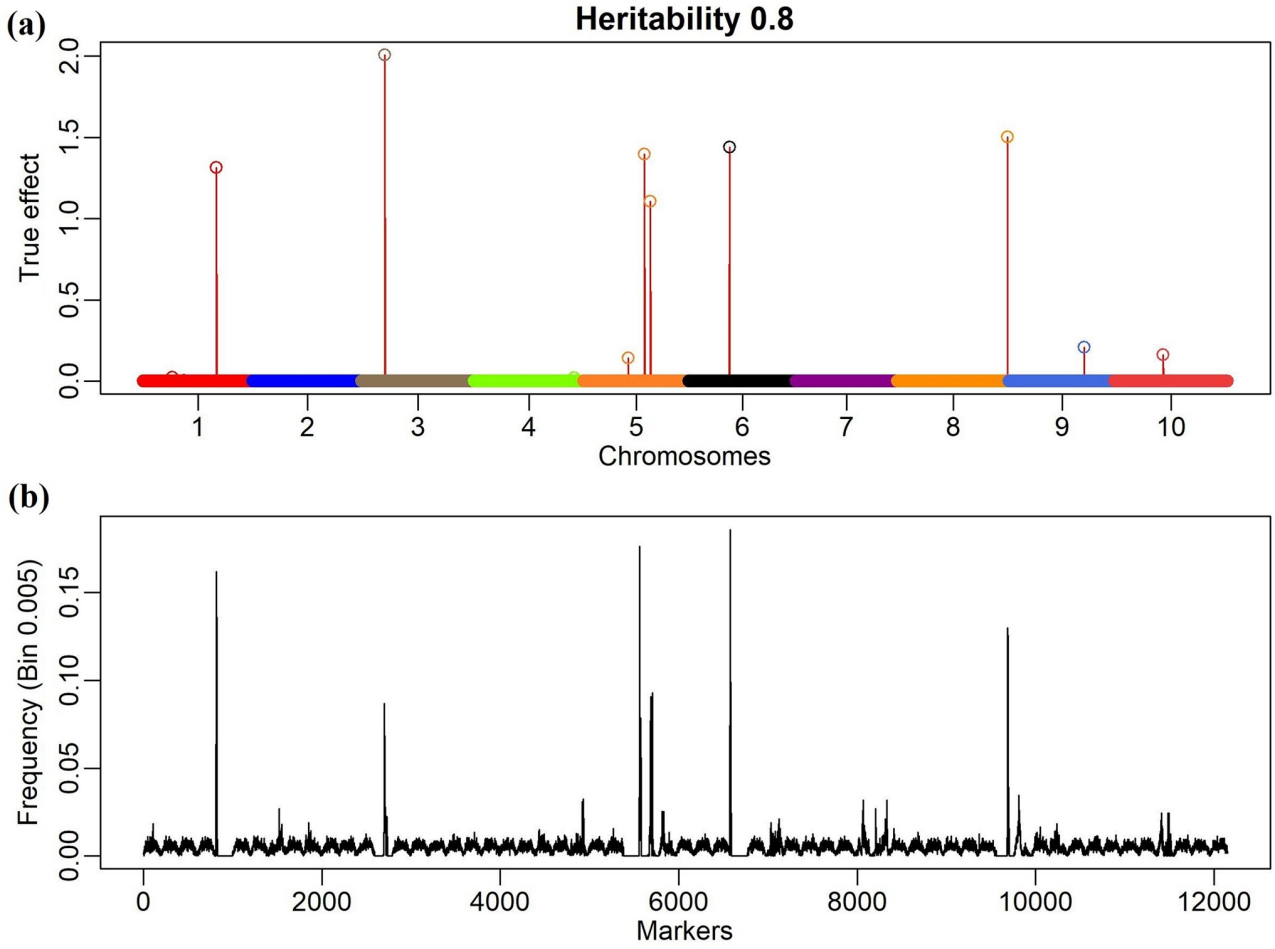
Simulated effects and relative frequency of the Bin 0.005 model. Panel A shows the absolute simulated effects. Panel B shows the relative frequency of the Bin 0.005 model for the heritability level of 0.8 in the Metropolis-Hastings algorithm.

The results that are shown in Fig 3 and Table 1 indicate that the number or size of the bins that divide the genome may influence the result of analyses, although the genomic profiles among the methods are not substantially divergent. One way to determine the number of bins is to use the LD as breakpoint information, to construct the so-called "natural bins" [9,14], where contiguous markers with high LD form an interval that presents similar information. However, given the stochastic nature of our model, it is expected for the breakpoints to be naturally built by the approximated function $f(\lambda |\mu ,\sigma_{e}^{2},\gamma ,\sigma_{\gamma }^{2},y)=P_{\lambda }$, which will converge to a uniform distribution *p*_*λ*_ ≐ {*λ*|*λ* ∊ *S* ≡ [*λ*_(min)_, *λ*_(max)_]} ≈ *q* under the scenario of blocks with high LD. To better understand this statement, consider Fig 4, which shows the scanning profile of *P*_*λ*_ across the simulated genome (with the QTL positions) for the model Bin 0.005 and a heritability level of 0.8.

**Fig 5:**
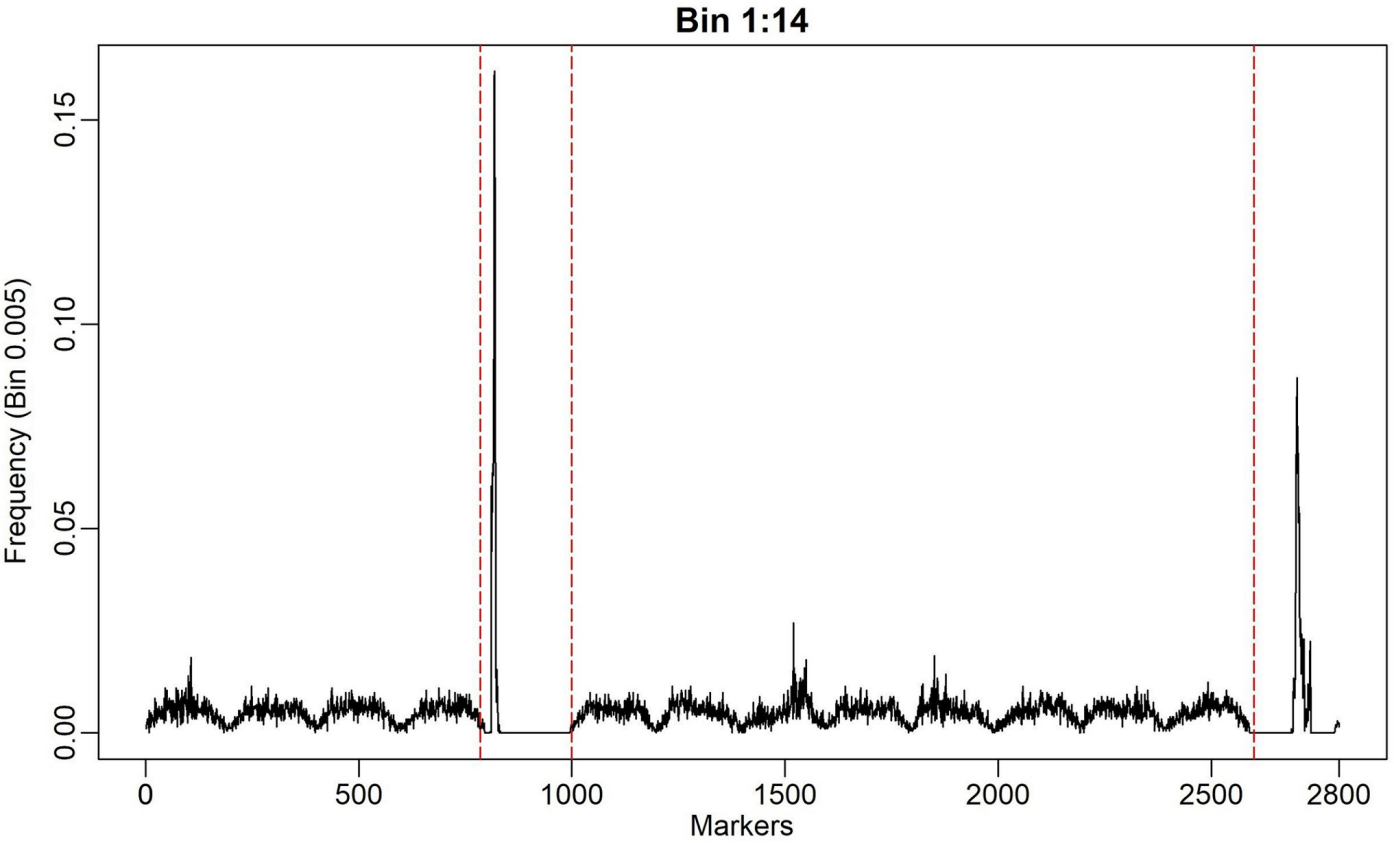
Relative frequency for the first 14 bins (Bins 1:14) of the Bin 0.005 model for the heritability level of 0.8. The dashed lines represent the possible natural bins that can be established instead of the first 14 bins, which are fixed a priori, with the Bin 0.005 model.

According to Fig 4, the algorithm runs through the genome randomly and with constant weight *P*_*λ*_ ≈ *q* until it identifies the signal of a possible QTL and generates a sharp peak. Moreover, high-frequency (or high-probability) points that are close to the main peaks are observed. The low-frequency points on the wavelets correspond to the established breakpoints and the pathway within these points is clearly uniform, as expected. Note that the positions of these bins in the genome are irrelevant since they are used only to optimize the stochastic search. However, as observed, the number of bins can influence the analysis in terms of speed and power of detection of true positive signals. For instance, the results in Fig 4 suggest possible natural bins (breakpoints) that could be established using sudden changes in the signals of the function $P_{\lambda_{j}}$. However, establishing these breakpoints without prior analysis may not be trivial. For better visualization, one can zoom in on the function $P_{\lambda_{j}}$ to examine the wavelet pattern of Fig 4 at specific points, as described in Fig 5.

In Fig 5, the wavelet function, which is represented by $P_{\lambda_{j}}$, for various bins in the Bin model 0.005 (200 markers per bin) is presented.

The artificial bins, at first glance, did not match with the true LD blocks. On the other hand, the stochastic searching was more effective in found the signal based on LD structure. Based on Fig 5, four bins could be established: the first appears before the first dashed red line in, the second between the first two dashed red lines, the third between the second and third dashed lines, and last after the last dashed line. The question arises of whether the candidate natural bins that are obtained through MCMC scanning have a biological interpretation i.e. matching with LD blocks.

**Fig 6:**
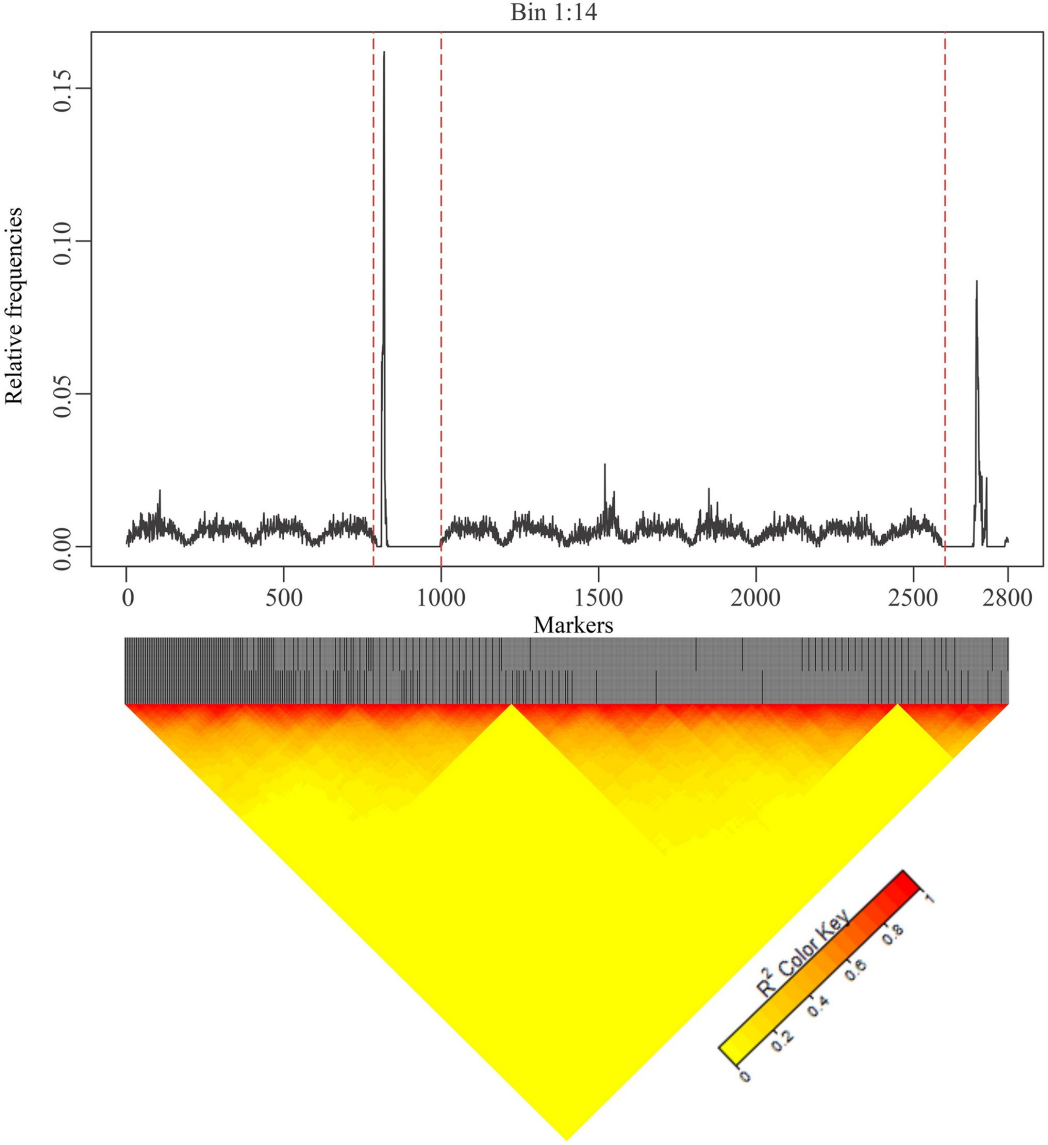
Genomic pattern and heat map of the first 14 bins obtained in the Bin 0.005 model. Panel A shows the genomic window related to the pattern on the first 14 bins obtained in the Bin 0.005 model. Panel B shows the heat map related to the disequilibrium pattern for correspondent genomic window. The closer to red, the greater the linkage disequilibrium.

To answer this question, a heat map was constructed for this range (Bin 1:14); the results are shown in Fig 6.

The heat map (Fig 6) has grouped the markers with high LD, in which the redder the markers cells are, the greater the linkage disequilibrium (in the same cluster). Thus, it is possible to observe that there are three blocks with high LD from Bin 1:14, i.e., two breakpoints and two possible natural bins. Thus, the LD pattern that is obtained through the heat map corroborates with the stochastic searching as illustrated in Fig 5 and suggest that the establishment of natural and artificial bins (on the contrary to the number of bins) can be irrelevant in functional models when using numerical algorithms to obtain $P_{\lambda_{j}}$.

**Fig 7:**
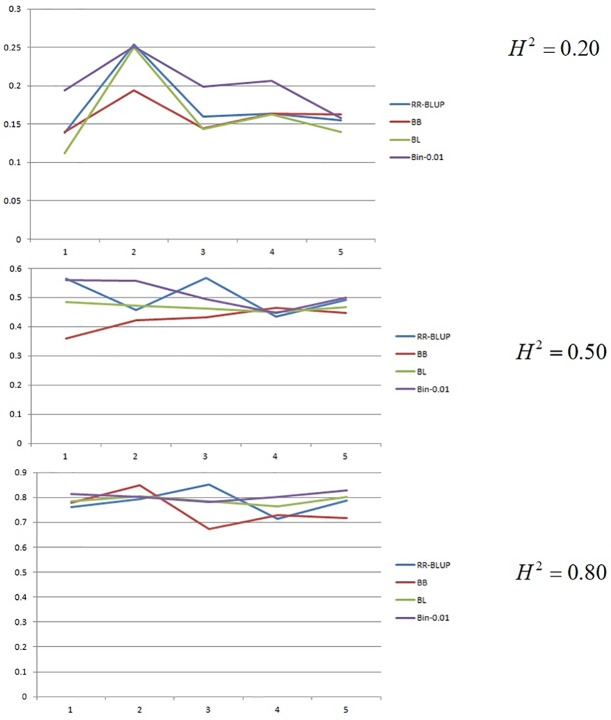
Predictive accuracy estimated through 5-fold cross-validation in simulated data at heritabilities 0.2, 0.5 and 0.8 for RR-BLUP (RR), Bayes B (BB), Bayesian Lasso (BL) and Bin 0.01 models.

The cross-validation results are shown in Fig 7. As observed in the prediction of true simulated values, the bin functional model showed better performance in predicting missing values in the low-heritability scenario. For the heritability level of 0.2, the bin model outperformed all methods and achieved the limit of prediction, namely, R^2^ = 0.201. At the heritability level of 0.5, RR-BLUP (R^2^ = 0.503) and the bin functional model (R^2^ = 0.511) were equivalent. This was also observed in 0.8 heritability level (R^2^ = 0.782 vs 0.805). Bayesian Lasso and Bayes B presented the following results for heritability levels of 0.2 (0.167 vs 0.165) and 0.8 (0.748 vs 0.787), respectively.

The same predictive pattern was observed for F_∞_ population in all scenarios. The similar pattern of *k*-fold results at heritability of 0.2 were observed being the best bin model achieving values of R^2^ of 0.21 vs 0.163; 0.164; 0.167 for RR-BLUP, Bayesian Lasso and Bayes B respectively. For the heritability of 0.5, the results were 0.509 vs 0.491; 0.487; 0.493 and for heritability of 0.8 the results were 0.782 vs 0.774; 0.763; 0.774 for bin model, RR-BLUP, Bayesian Lasso and Bayes B respectively.

### Predictive results in *Eucalyptus* data

**Fig 8:**
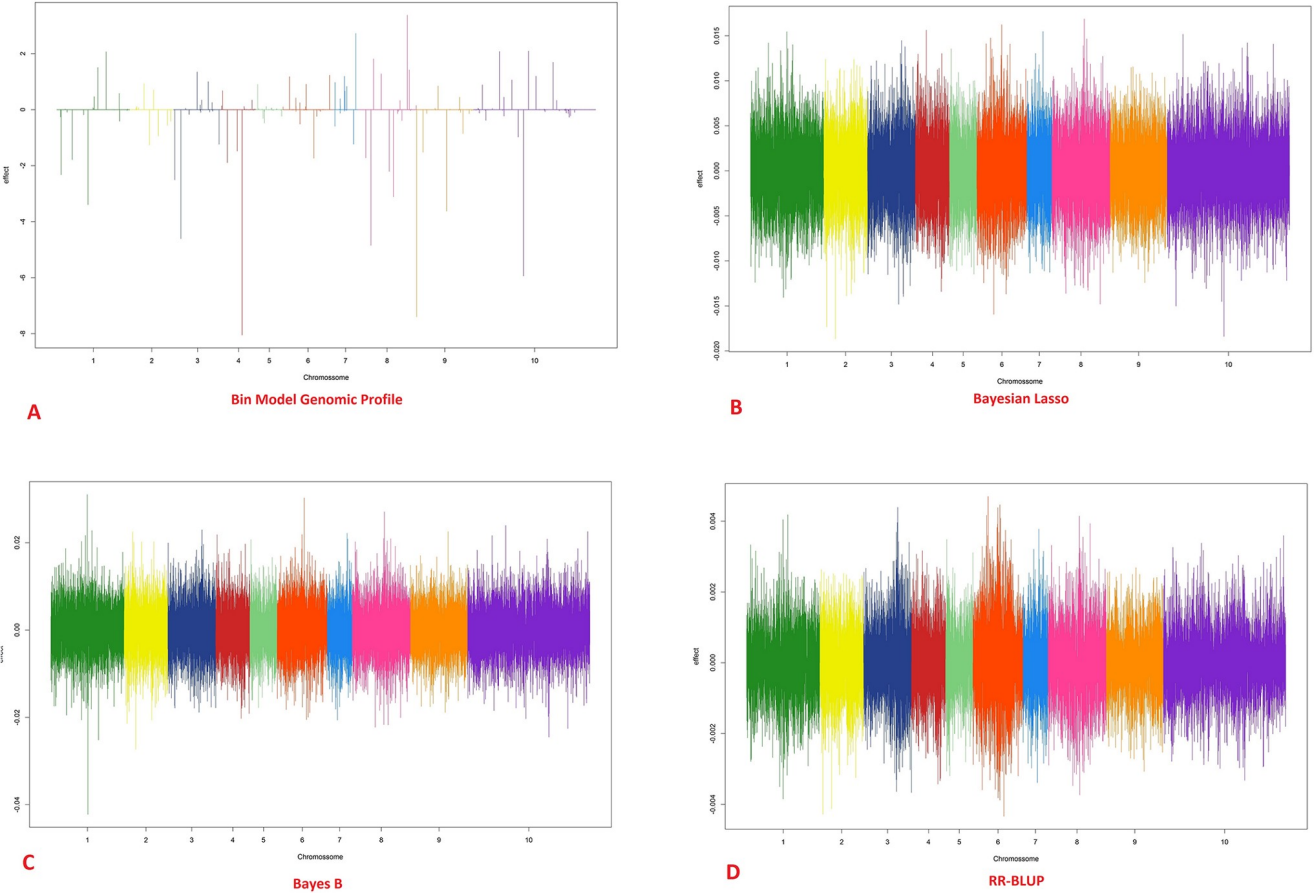
Genomic profile of 611 *Eucalyptus* plants genotyped with 36,812 SNPs obtained from the models. (A) Bin 0.01, (B) Bayesian Lasso, (C) Bayes B and (D) RR-BLUP.

The cross-validation results obtained from *Eucalyptus* data indicate that the bin model was equivalent to the comparison methods (RR-BLUP, Bayes B and Bayesian Lasso). The average R^2^ values that were estimated by considering all folds were 0.30, 0.28, 0.286 and 0.283 for Bin model, RR-BLUP, Bayes B and Bayesian Lasso, respectively. The genome patterns of marker effects are given in Fig 8. In general, the bin functional model presented a higher-resolution pattern of marker effects than the comparison methods, thereby indicating its potential as a prediction method.

**Fig 9:**
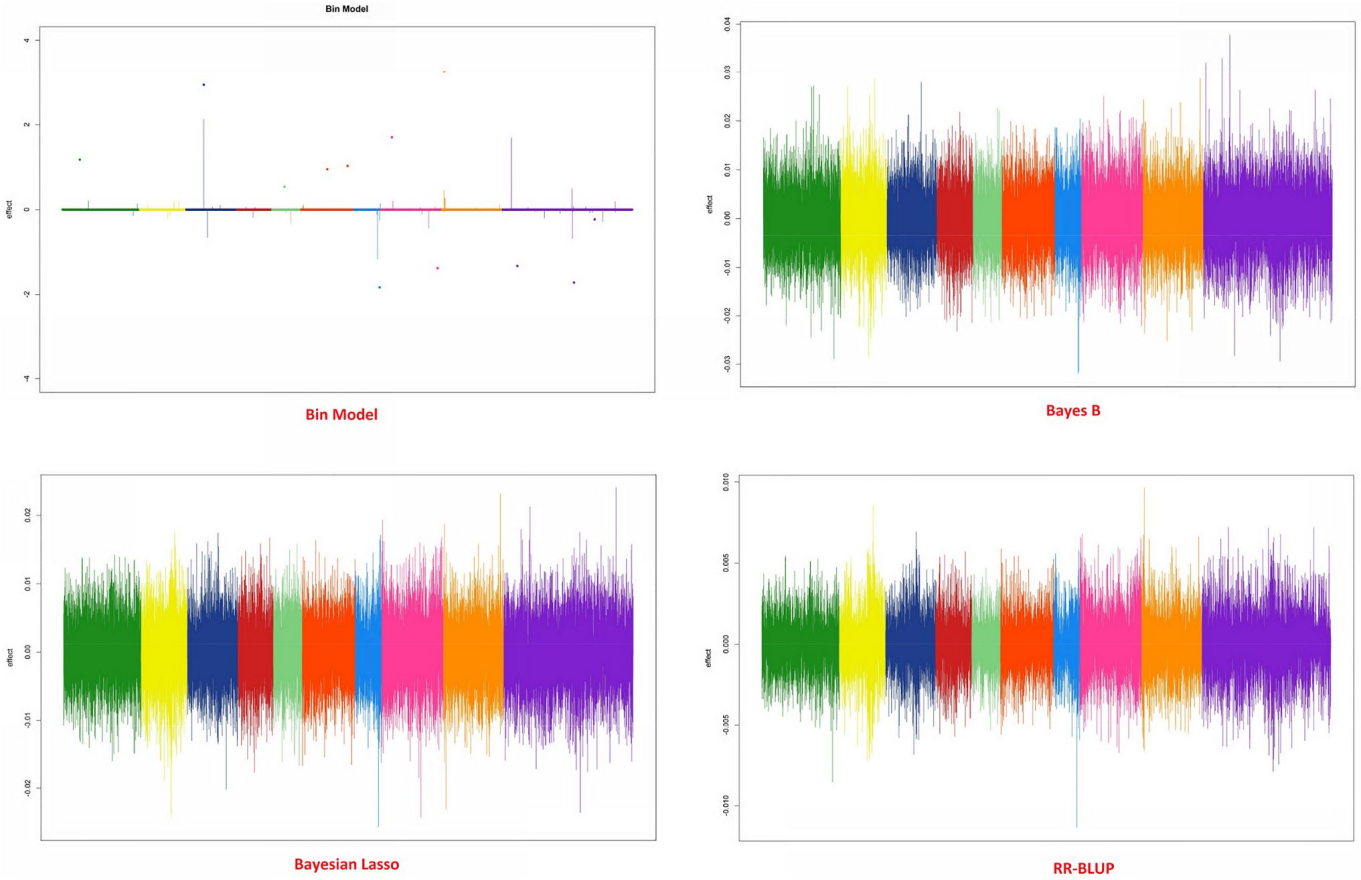
Genomic profile relates to 611 *Eucalyptus* plants simulated from the 36,812 SNPs obtained the real data. The models used were the Bin 0.01, Bayesian Lasso, Bayes B and RR-BLUP models. The dots represent the position e effects of simulated genes.

Using the Eucalyptus genome structure to simulate the genes, again the superiority of bin model on the concurrent models is evident. In all scenarios, the bin model was superior in ranking and predicting the simulated genotypic values (Table 1). The LD pattern of causal regions and the genomic pattern can be observed at Fig 9.

**Fig 10:**
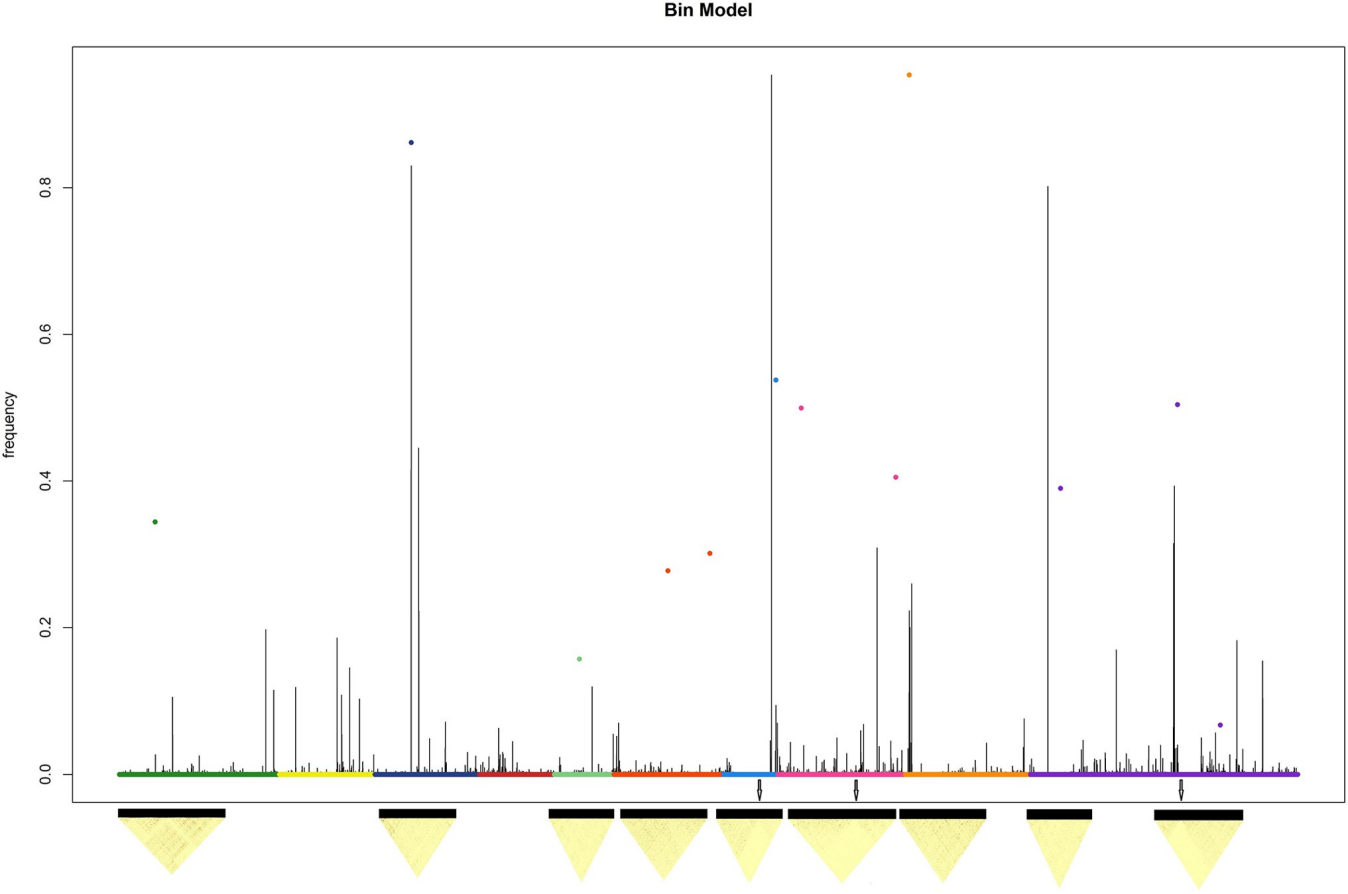
Weighted function describing the Genomic profile relates to 611 *Eucalyptus* plants simulated from the 36,812 SNPs obtained the real data. The dots represent the position e effects of simulated genes. The heat map represents the genomic LD around the simulated QTLs.

In the Fig 9, as also observed in the true data, the best resolution of the genomic structure is evident. But, for the simulated data the best genome resolution converged to best predictions (Table 1). Also, plotting the weight matrix $P_{\lambda_{j}}$ vs. LD pattern, it was revealed the robustness of method to deal with different LD structures since the model was able to find causal region even in LD window showing in low LD blocks where natural bins are absents (Fig 10).

## Discussion

### Functional models and computational efficiency

A genome panel with a high density of markers that presents high linkage disequilibrium contains several redundant markers that do not provide any new information and may lead to multicollinearity problems in the high-dimensional models that are used in the genomic selection framework, thereby generating statistical and computational challenges. Xu [9] claims that the current genome-wide analysis is tailored to the opposite situation to the previously observed situation in QTL mapping, i.e., in QTL mapping methods, the objective was to saturate the genome with pseudo-markers that are based on a small number of pivotal markers, whereas the present situation requires eliminating markers that have the same amount of information.

It is clear that regression models of marker-specific variance, such as those that are used in the Bayesian alphabet [8], present problems in large panels, which are associated with limited phenotypic information, i.e., the larger the marker panel, the greater the computational demands in the estimation of marker effects and the more severe the multicollinearity problems. Hu et al. [12] proposed the continuous genome model, which uses a genomic functional approximation through artificial bins and reference markers as breakpoints in searching for a low-dimensional representation of the infinitesimal model.

In the present study, we proposed a more direct approach of functional models and numerical integration of the genomic function and relaxed the assumption of high-LD blocks in the bins. This enabled a type of weighted mean across the bins to be obtained instead of an average effect under a Haar wavelet assumption on the marker intervals [12]. We sought to find empirically a weighted function that describes the marker effect based on its genomic position. In this sense, the current bin approach is very similar to the knots that are used in spline regressions since various piecewise functions can be obtained by the flanking markers. In other words, instead of fitting a polynomial spline within each knot [27] we sought to build an empirical function that visits the candidate markers and their genomic positions using the Metropolis-Hastings algorithm. We allow ourselves to use the terms “bins” and “knots” synonymously since in the functional spline model, each knot represents a piecewise polynomial function that is delimited by breakpoints [27].

In general, the assumption of a Haar wavelet (1 0–1) is based on the previous establishment of natural bins, which are founded on breakpoint markers that present an objective justification (e.g., high-LD blocks) [28]. However, the size of natural bins may vary depending on the genetic background, population crossing structure and sample size. For instance, introducing (or deleting) an individual in the current sample can produce new breakpoints; thus, new bins may be established. Therefore, in this study, we prefer to use the so-called artificial bins or knots instead of the biological breakpoints since the number of knots does not depend on the sample [9].

In all LD scenarios used here, the bin model outperformed the candidate models. These results agree with our initial assumption that the providing natural bins, based on LD, are not necessary in continuous genome if stochastic searching is performed. On this framework, it was allowed that the bin model identified true spike pattern where the other models were efficient only on high heritability scenarios (Figs 2 and 3 and S10 and S11 Figs). Therefore, the number of bins or knots, in our context, no longer depends on where the bins will be established, but on the number of bins to be used, thereby making the accuracy in the fitting of *p*(*λ*|*y*, *γ*) and *γ*(*λ*) equal to the computational demand.

In the scenarios described above, the number of bins was selected to accelerate the regression by minimizing the computational demand. For instance, the model bin 0.001 was fitted using only 12 markers per round of MCMC, which required a computation time that was practically insignificant given the available computational resources. It was approximately 3500 times faster than the full model. Since the library BGLR is compiled using Fortran and C, it very difficult to compare the computational performance with S language used in R. But, using one round of MCMC for each candidate model on S language, the bin model with 12 markers outperformed the Bayesian RR-BLUP, BayesB and LASSO in 2100, 3050 and 3750 times respectively.

The requested computational demand is inversely related to the number of bins, i.e., the smaller the number of bins, the faster the MCMC process and lower the computational demand. For instance, the model Bin 0.001 was 3000 times faster than the model Bin 0.1. Furthermore, model Bin 0.001 (1000 markers per bin) achieved the best predictive ability among all evaluated bin configurations and multiple traditional full regression models of genomic selection. This comparison was done using a workstation Intel^®^ Xeon^®^ E5 20 core 80 GB 2TB.

### Functional models predictive ability

The predictive ability of the Bayesian functional model, in most settings, was higher than those of the RR-BLUP, Bayes B and Bayesian Lasso models (Table 1). This corroborates the results of [9,12] and can be explained by the smoothing effect on markers’ collinearity and the strong shrinkage problems that emerge from the model constraints. Taking this into account, the functional model reduced the shrinkage effect in the true QTL, which was reflected by the high-resolution scanning of the genome and the lower MSE value [12]. Thus, problems that are related to the downwardly biased estimation that is observed in shrinkage methods [11] in true positives effects may be avoided.

If *K* → *M* (e.g., the number of bins is equal to the number of markers), the bin model converges to the classical regression method of the Bayesian alphabet (best Xu’ model for human’s data). Thus, in addition to the three bin configurations that are shown above, other scenarios with more bins were tested (e.g., bins of 10 markers). However, an increase in the predictive accuracy of the simulated true values was not observed; only an increase in computational demand was observed converging to predictive values that were near the values that were obtained by the full regression model (0.678, 0.838 and 0.902 for heritability levels of 0.2, 0.5 and 0.8, respectively). This result indicates that the bin model can outperform the original full marker model in terms of estimation and predictive ability. However, this advantage, as observed in spline functional models, depends on the correct setting of knots across the genome.

The bin model outperformed the comparison models in predicting missing values in scenarios of simulated unbalance. In three heritability scenarios, the bin model outperforms all comparison models, which suggests its applicability in GS to predicting untested genotypes in different LD structures. As in true value prediction, the higher the simulated heritability, the more similar are the predictions among the GS methods. Equivalent results were observed by [9], although the model performance was not evaluated for different unbalance scenarios and heritability levels.

It is not our intention in this study to give a detailed description of the functional model in *Eucalyptus spp*., but instead to use these data to illustrate how the bin model performs on various populations and LD structures. Instead to those observed in F_2_ population, high LD blocks in *Eucalyptus* were not evident (Fig 10). Only three LD blocks were observed in the chromosome 7, 8 and 10 in genomic regions near to simulated QTLs. In these circumstances, the stochastic walking was not very evident as in F_2_ high LD blocks. The low LD disequilibrium can be attributed to mixture population involving four eucalyptus species: *E*. *grandis*, *E*. *urophylla*, *E*. *globulus* and *E*. *camaldulensis*. Even in this circumstances, the $P_{\lambda_{j}}$ was effective in searching for candidate regions and helped to obtain a high resolution in genomic profile (Fig 9). The individual pattern of LD blocks is available in supplemental material (S1–S9 Figs).

The results of the cross-validation study along with the pattern of the marker effect throughout the genome indicate the power of the method in predicting missing values and depicting a more realistic pattern of marker effects across the genome, where several markers exhibit some effect but the majorities do not pay a role in the genomic architecture. The difficulty of BB, RR and BL in presenting a realistic picture of the distribution of the genetic effects for circumference at breast height (CBH) was evident, since all markers seem to contribute equally to this trait.

The predictive differences in eucalyptus using the observed phenotypic simulated values can be the indicative that the genomic architecture can influence the predictive results. Whereas in the Fig 9, few peaks were observed due the number of QTLs simulated, in the Fig 8 several candidate peaks were observed suggesting several QTLs controlling the CBH. In the other hand, the genomic profile given by the other models did not present great differences even in polygenic structures (Figs 8 and 9). Since the true position of QTL for the real data is not known, it is very complicate to infer about the superiority of the bin model on the other models in long- run selection. However, the simulated results associated to LD pattern in eucalyptus suggested that bin model in polygenic structures could be very attractive to genomic selection in long-run even in low LD populations.

The choice of the most suitable model for use in genome analysis may depend on several interconnected attributes, such as the genetic architecture (e.g., number of genes, LD decay, heritability, and the presence of dominant and other non-additive effects), population structure (e.g., sample size and pedigree), marker density and number of phenotyped individuals [12,14,16,29,30]. According to Su et al. [16], major QTL for traits with low heritability and polygenic traits, even those that are highly heritable, are often difficult to detect in high-dimensional shrinkage models.

Daetwyler et al. [29] note that the efficiency of each method depends on the genetic architecture and simulated scenario. Under these circumstances, the model assumptions are an important factor. For instance, in polygenic or infinitesimal scenarios, the GBLUP (or RR-BLUP) model is likely to have better predictive capacity compared to Bayesian regression models of marker-specific variance. Under the simulated scenario considered in this study (polygenic architecture with SNP effect that is sampled from a Gaussian distribution with zero mean and common variance), the RR-BLUP model, which assumes the same prior hyperparameters, could be favored. However, the RR-BLUP Gaussian prior, with homogenous variance, did not favor the model uniformly across the prediction scenarios and this method outperformed the Bayesian models only for the heritability level of 0.2.

Based on this argument, it could be imagined that the Bin model would present lower predictive ability under the infinitesimal scenario since our functional model uses stochastic search, which tends to select markers that have greater effect and penalizes to zero those markers that are not included in the current MCMC process. To include markers that exhibit minor effects, the functional model could be fitted using many bins; however, it will converge to the computational problems that were previously discussed. Another problem is that the true genetic architecture is unknown and in incorrect "guess" about the number of bins may decrease the Bin model’s performance relative to RR-BLUP or GBLUP (the “no free lunch” theorem). It is evident that in a polygenic/infinitesimal scenario with a Gaussian stochastic process with common variance, RR-BLUP may present better predictive ability; however, the advantage of the bin methods relies on their asymptotic property when *K* → *M*. In a polygenic/infinitesimal scenario, the GBLUP random regression model (which is theoretically equivalent to RR-BLUP) is more accurate than Bayesian regression models; conversely, these Bayesian models, have presented advantages in the oligogenic or polygenic scenarios [29].

According to Su et al. [16], a bin is defined as a linkage disequilibrium (LD) block in which all SNPs are identically segregated. According to Fig 4, it is possible to construct causal disequilibrium blocks naturally during the genomic search since, as was emphasized above, if there is high linkage disequilibrium among markers in a genomic window, *p*(*λ*|*γ*) will be uniform. In addition, if the phenotypic vector *y* provides no information on *p*(*λ*|*γ*), the distribution *p*(*λ*|*γ*) converges to a discrete uniform distribution throughout the bin where *λ* is allocated. Thus, the functional model, using stochastic search, seeks to describe LD bins naturally (as natural knots) and could be used in a two-stage analysis. Similar to an *ad hoc* procedure, it could perform the initial scanning with an arbitrary number of bins by capturing the information from *p*(*λ*|*γ*) and establish breakpoints to be used in a further analysis, similar to that proposed by Xu [9]. The high-frequency signals of *p*(*λ*|*γ*) are candidates for natural breakpoints (but not LD blocks limits as discussed above), as used by Xu [9], i.e., one can use artificial bins to automatically obtain natural bins.

Yu et al. [14] argue that the precise estimation of the LD breakpoints depends on the marker density in a specific genomic window: the breakpoints can be identified more accurately in a higher-marker-density scenario. In *Eucalyptus*, some LD blocks were observed in the genomic window related to simulated QTL, but not in high LD (Fig 10). In these circumstances, the establishment of artificial bins will be very difficult and the uniform searching pattern showed in F_2_ was not observed. Therefore, one could understand that our model might be more robust to be applied in different LD structures than bin model proposed by Xu [9] as showed in humans and eucalyptus results where the bin model was not the best one requesting the whole SNP panel to increase the accuracy.

Since the objective in several genomic studies is to detect candidate genes that may control quantitative traits, functional models that are based on the artificial bin methodology may be a good tool for studying the genetic architecture of traits. Thus, given the genomic profile that was obtained in Figs 2, 8 and 9, functional models could be easily applied in genome-wide association studies (GWAS) to identify candidate regions. It is because the penalized posterior $\underset{\gamma \in ℜ}{\text{arg}.\text{max}}\left[p(\gamma |y)\right]p(\lambda |\gamma ,y)$ does not show strong shrinkage effect, such as that observed in the penalized models allowing statistical tests on the markers effects. For example, recently, Wu et al. [31] performed a study on constructing haplotypes (similar to the natural bins) in the bovine genome to detect candidate genes that affect traits that are related to meat quality. According to these authors, most significant QTL that were detected by individual SNP analysis were also identified using the haplotype-based analysis. Other bins/ haplotypes models has been proposed, including those assuming convolution of Gaussian distribution supposing independency among haplotypes blocks in GWAS [32]. Given that haplotypes are supposed as independent the GS could be applied on the piecewise kernels of the Gaussian in order to predict the genotypic values since the ML estimators are equivalent to independent multiple regression. The problems with these models in GWS are related with identificability i.e., the sum of “independent” bins predictors distributed across the kernel of multiplied Gaussian does not match with the mean of the resulted likelihood and the model expectation plus variance are not more linear. While this might be a problem in GWS framework, it is not for GWAS as proposed by the authors. Our model keeps the model identifiability since the penalty $P_{\lambda_{j}}$ allows that all markers contribute to predict the genomic values and convolution or multiplication of Gaussian is not necessary.

The main advantage of applying the bin method that is associated with functional models in genomic selection (GS) is that it places the GS models back in the genetic setting since the marker positions and LD information are again taken into account in the search for causal regions, whose identification may contribute to the accurate prediction of genomic values. Additionally, any penalized regression model or the Bayesian alphabet framework can be adapted to the bin model, thereby enabling a fast analysis. Thus, since this methodology captures the gene pattern independent of LD blocks, this approach is expected to be more efficient than parametric/semi-parametric predictive machinery since the long-term success of genomic selection depends on the relationship between causal regions and the presence of LD blocks.

Based on the results that are described above, it is inferred that Bin models are a potential tool for the prediction of genomic values. This approach unifies the predictive and QTL searching models for depicting the genetic architecture based on the functional relationship between the effect of an SNP and its position. For the set of simulated scenarios, the proposed method showed advantages over the RR-BLUP, Bayes B and Bayesian Lasso methods in predicting genomic values and missing phenotypic information. Although the predictive ability among the models was similar for *Eucalyptus* real data, the bin functional model achieved high-resolution scanning of the genome for causal regions, thereby highlighting the potential of the method for use in both prediction and genomic association studies.

## Supporting information

S1 Dataset Genomic dataset. (ZIP)

S1 File R-package. (ZIP)

S1 Text Additional model justification. (DOCX)

S1 Fig Genomic window build from 900 SNPs around the QTL simulated on the first chromosome. (JPEG)

S2 Fig Genomic window build from 1000 SNPs around the QTL simulated on the third chromosome. (JPEG)

S3 Fig Genomic window build from 1000 SNPs around the QTL simulated on the fifth chromosome. (JPEG)

S4 Fig Genomic window build from 2000 SNPs around the two QTLs simulated on the sixth chromosome. (JPEG)

S5 Fig Genomic window build from 1000 SNPs around the QTL simulated on the seventh chromosome. (JPEG)

S6 Fig Genomic window build from 3100 SNPs around the two QTL simulated on the eighth chromosome. (JPEG)

S7 Fig Genomic window build from 2000 SNPs around the QTL simulated on the ninth chromosome. (JPEG)

S8 Fig Genomic window build from 1000 SNPs around the first QTL simulated on the tenth chromosome. (JPEG)

S9 Fig Genomic window build from 1500 SNPs around the two last QTL simulated on the tenth chromosome. (JPEG)

S10 Fig Absolute simulated actual effects of QTL along the human’s genome and absolute estimates from methods rr-BLUP (RR), Bayes B (BB) and Bayesian Lasso (BL). (JPG)

S11 Fig Absolute simulated actual effects of QTL along the human’s genome and absolute estimates from the methods for Bin 0.001, Bin 0.005 and Bin 0.01. (JPG)
