# Supplementary material for: Functional models in genome-wide selection
Source: PLoS One. 2019 Oct 23;14(10):e0222699. doi: 10.1371/journal.pone.0222699 (PMC6808424; doi:10.1371/journal.pone.0222699)
Supplement: S1 File — (ZIP) [file pone.0222699.s002.zip › BFBM/html/00Index.html]

R: Bayesian Functional Bin Model

# Bayesian Functional Bin Model

---

## Documentation for package ‘BFBM’ version 1.0

- DESCRIPTION file.

## Help Pages

|  |  |
| --- | --- |
| BFBM-package | Bayesian Functional Bin Model - BFBM |
| bayesbinmod | Function bayesbinmod |
| BFBM | Bayesian Functional Bin Model - BFBM |
| effect | \*effects\* |
| GBV | \*GBV\* |
| markers | \*markers\* |
| phenotype | \*phenotype\* |
