# Supplementary material for: Functional models in genome-wide selection
Source: PLoS One. 2019 Oct 23;14(10):e0222699. doi: 10.1371/journal.pone.0222699 (PMC6808424; doi:10.1371/journal.pone.0222699)
Supplement: S1 File — (ZIP) [file pone.0222699.s002.zip › BFBM/html/bayesbinmod.html]

R: Function bayesbinmod

|  |  |
| --- | --- |
| bayesbinmod {BFBM} | R Documentation |

## Function bayesbinmod

### Description

This is the function for genomic value prediction using Bayesian Functional Bin Model.

### Usage

```
bayesbinmod(x = x, y = y, nIter = nIter, nburn = nburn, nthin = nthin, size_bin = size_bin)
```

### Arguments

|  |  |
| --- | --- |
| `x:` | Markers genotype matrix (e.g., {aa, Aa, AA} = {0, 1, 2}) of dimensions nobs\*nvars; each row is a observation vector of an individual and each column is a genotypic indicator vector for a marker. |
| `y:` | a vector of response variable (phenotypic observations), of dimensions *nobs*\*1. |
| `nIter:` | Number of iterations of interest for the final MCMC sample. It is a combination between *(nIter + nburn)\*nthin*. |
| `nburn:` | The number of burn that must be performed to obtain the MCMC sample. |
| `nthin:` | Number of jumps that must be performed between the observations to obtain the MCMC sample. |
| `size_bin:` | Bin size to be considered in the analysis, in percentage. Enter the percentage of the quantity of the markers in the bin. The bins will have the same amount of markers. |

### Details

The function implements the MCMC by Gibbs sampling for a Bayesian functional model and it creates an MCMC sample of the genotypic effect that will be saved in the directory in .txt format.

### Value

A matrix of dimension nIter by ncol(x).

### Author(s)

Ernandes Guedes Moura, Andrezza Kellen Alves Pamplona and Marcio Balestre.

### References

MOURA, E. G. Aplicacao de modelos funcionais na selecao genomica ampla. 2017. 54 p. Dissertacao (Mestrado em Estatistica e Experimentacao Agropecuaria) - Universidade Federal de Lavras, Lavras, 2017.

### Examples

```
### Load the demo data
data(markers)        ### markers genotypic matrix.
data(phenotype)      ### phenotypic vector.
data(GBV)            ### Genomic breeding values.
data(effects)        ### markers effects matrix.

y <- phenotype
Z <- markers

### Perform the analysis under the default settings
BF.model <- bayesbinmod(x = Z, y = y, nIter = 11, nburn = 1, nthin = 1, size_bin = 0.01)

md  <- BF.model$Beta   ### chain of the estimated mean.
eff <- BF.model$A      ### chain of the estimated markers effects.
va  <- BF.model$Va     ### chain of the estimated markers variances.
ve  <- BF.model$Ve     ### chain of the estimated residual variance.
Wa  <- BF.model$Wald   ### chain of the Wald test.

### Return the mean of the effects of each marker.
media  <- colMeans(eff)   

### Return the Genomic Estimated Breeding Values (GEBV)
vgg.hat <- Z %*% media

### Return the predictive accuracy of the model
accuracy <- cor(GBV,vgg.hat)   

### Plot true and estimated effects
par(oma <- c(0.5, 1, 0, 1), mar=c(2.3, 2.3, 1.5, 0), mgp = c(1.4, 0.5, 0))
plot(unlist(effects),type = "o",xlab = "",ylab = "",ylim=c(min(effects),max(effects)))
par(new=T)
plot(media,type = "l",xlab = 'Marcas',ylab = 'Effects',col = "red",ylim=c(min(effects),max(effects)))
legend(1900,-0.3,c("True effects","Estimated effects"),col=c("black","red"),lty=1)
box()
```

---

[Package *BFBM* version 1.0 Index]
