# Supplementary material for: Functional models in genome-wide selection
Source: PLoS One. 2019 Oct 23;14(10):e0222699. doi: 10.1371/journal.pone.0222699 (PMC6808424; doi:10.1371/journal.pone.0222699)
Supplement: S1 File — (ZIP) [file pone.0222699.s002.zip › BFBM/html/bayes_binmod_effects.html]

R: \*effects\*

|  |  |
| --- | --- |
| bayes\_binmod\_effect {BFBM} | R Documentation |

## **effects**

### Description

Markers effects matrix to compute the model accuracy. A matrix with the effects of the 2448 markers, in which 12 markers have non-zero effects.

### Usage

```
data(effects)
```

### Format

effects data. int [1:2448, 1] 0 0 0 0 0 0 0 ...

### Examples

```
### Load example of markers effects data
data(effects)

plot(c(1:nrow(effects)),unlist(effects))
```

---

[Package *BFBM* version 1.0 Index]
