# Supplementary material for: Functional models in genome-wide selection
Source: PLoS One. 2019 Oct 23;14(10):e0222699. doi: 10.1371/journal.pone.0222699 (PMC6808424; doi:10.1371/journal.pone.0222699)
Supplement: S1 File — (ZIP) [file pone.0222699.s002.zip › BFBM/html/bayes_binmod_GBV.html]

R: \*GBV\*

|  |  |
| --- | --- |
| bayes\_binmod\_GBV {BFBM} | R Documentation |

## **GBV**

### Description

This is the Genomic breeding value (GBV) of the 300 individuals.

### Usage

```
data(GBV)
```

### Format

phenotypic data. int [1:300,1] 0.5006 -0.0776 1.2612 2.9840 2.4163 ...

### Examples

```
### Load example of GBV data
data(GBV)
```

---

[Package *BFBM* version 1.0 Index]
