# Supplementary material for: Functional models in genome-wide selection
Source: PLoS One. 2019 Oct 23;14(10):e0222699. doi: 10.1371/journal.pone.0222699 (PMC6808424; doi:10.1371/journal.pone.0222699)
Supplement: S1 File — (ZIP) [file pone.0222699.s002.zip › BFBM/html/bayes_binmod_markers.html]

R: \*markers\*

|  |  |
| --- | --- |
| bayes\_binmod\_markers {BFBM} | R Documentation |

## **markers**

### Description

This is the genotypic matrix {aa, Aa, AA} = {0, 1, 2} of 2,448 SNP markers, in 300 individuals of an *F\_{2}* population, distributed in ten chromosomes of 120 cM each one.

### Usage

```
data(markers)
```

### Format

genotypic data. int [1:300, 1:2448] 1 1 1 2 2 1 1 2 1 1 ...

### Examples

```
### Load example of genotypic markers data
data(markers)
```

---

[Package *BFBM* version 1.0 Index]
