# Supplementary material for: Functional models in genome-wide selection
Source: PLoS One. 2019 Oct 23;14(10):e0222699. doi: 10.1371/journal.pone.0222699 (PMC6808424; doi:10.1371/journal.pone.0222699)
Supplement: S1 File — (ZIP) [file pone.0222699.s002.zip › BFBM/html/bayes_binmod_phenotype.html]

R: \*phenotype\*

|  |  |
| --- | --- |
| bayes\_binmod\_phenotype {BFBM} | R Documentation |

## **phenotype**

### Description

This is the phenotypic data of 300 individuals.

### Usage

```
data(phenotype)
```

### Format

phenotypic data. int [1:300,1] 0.9918 2.6173 -0.8406 0.8070 2.4738 ...

### Examples

```
### Load example of phenotypic data
data(phenotype)
```

---

[Package *BFBM* version 1.0 Index]
