# Supplementary material for: Functional models in genome-wide selection
Source: PLoS One. 2019 Oct 23;14(10):e0222699. doi: 10.1371/journal.pone.0222699 (PMC6808424; doi:10.1371/journal.pone.0222699)
Supplement: S1 File — (ZIP) [file pone.0222699.s002.zip › BFBM/html/BFBM-package.html]

R: Bayesian Functional Bin Model - BFBM

|  |  |
| --- | --- |
| BFBM-package {BFBM} | R Documentation |

## Bayesian Functional Bin Model - BFBM

### Description

Package for genomic selection using bayesian functional bin model.

### Details

The DESCRIPTION file:

|  |  |
| --- | --- |
| Package: | BFBM |
| Type: | Package |
| Version: | 1.0 |
| Date: | 2019-01-05 |
| License: | GPL (>= 3) |
|  |
|  |

### Author(s)

Ernandes Guedes Moura, Andrezza Kellen Alves Pamplona and Marcio Balestre.

Maintainer: <ernandes.moura@ifma.edu.br>

---

[Package *BFBM* version 1.0 Index]
