# Supplementary material for: Functional models in genome-wide selection
Source: PLoS One. 2019 Oct 23;14(10):e0222699. doi: 10.1371/journal.pone.0222699 (PMC6808424; doi:10.1371/journal.pone.0222699)
Supplement: S1 Text — (DOCX) [file pone.0222699.s003.docx]

**S1 Text**

**Model justification**

Using the genomic relationship described in the Fig 1, one can figure out a hypothetical mathematical function $f(\lambda)$ able to map important regions in each chromosome at genome whose output is related with the additive effect $\gamma$ in each genome position. In other words, the relationship between the position scanning and the candidate regions may be given by a unknown function $f(\lambda)=\gamma$, where $\lambda$ is the position in base pairs or centiMorgan (cM) in the genome and $\gamma$ is a continuous vector on each chromosome, , that is related to the additive effect of a candidate gene at the specified position $\lambda$. Here it will described the functional relationship $f(\lambda)=\gamma$ by $\gamma(\lambda)$, i.e., a function that describes the additive effect $\gamma$ of the gene under the scanning of the genome position$\lambda$. However, $f(\lambda)$ and $\gamma$ are unknown in genome selection context where the only information available are those related to a phenotype of an individual (*y*), the SNP genotype matrix (**Z**) frequently coded as (2,1 and 0) and sometimes the SNPs positions $\lambda$ on the genome.

Looking the Fig 1, it can be established the domain of the unknown genomic function in which is given by $\gamma(\lambda):=\left\{\lambda|\lambda\in\Omega\equiv\left[1,L\right]\right\}$ within a specific genome, where *L* is the chromosome length of any species.

As showed by Xu [9] the integration of $\gamma(\lambda)$returns the predictive additive value (VGA) of the i-th individual given that in classical genomic selection model $VGA_{i}=\sum_{j=1}^{p} z_{ij}\hat{\gamma}_{j}$where *p* is the size of SNP panel. It is evident that$\lim_{p\to\infty} \left(\sum_{i=1}^{p} z_{ij}\hat{\gamma}_{i}\right)=\int z(\lambda)\hat{\gamma}(\lambda)d\lambda$where th discrete genome could be converted in a continuo genome.

In functional model the phenotypic vector $\mathbf{y}$may be obtained by the integration of $\gamma(\lambda)$ [9,20], changing the domain of $\gamma(\lambda)$ to the domain of *y* It can be performed using markers (SNP) genotype matrix $\mathbf{Z}$where the genome position $\lambda$ for each marker is known $\left[\mathbf{Z}(\lambda)\right]$. This is known in functional model theory as scalar response for an integrated functional model [21] where ; in other words, the integration of additive curve (Fig 1) could be approximated by a piecewise sum of functional basis **B** of *q* order (see [21] for more details). However, in this study, we propose to use the ordered SNP matrix as a dosage effect to integrate the function $\gamma\left(\lambda\right)$ instead a functional basis only.

The above assumption allows the empirical integration $\gamma(\lambda)$ even that this function be unknown. In other words, assuming$f(\lambda)=\gamma$i.e. the continuo additive effect could be described by a functional relation with their position in the genome, the functional models with scalar response, multiplying by the ordered SNP matrix in both equation sides we have the following equality: $\overbrace{\mathbf{Z}(\lambda)f(\lambda)}^{Continuos aproximation of Genome}=\overbrace{\mathbf{Z}(\lambda)\gamma}^{Discrete Genome in Classical GS }$. Taking $\mathbf{Z}(\lambda)\gamma=\widehat{VGA}$ as the classical linear prediction of additive genomic value $\widehat{VGA}$in genomic selection models (GS), i.e., the linear combination of the SNP state (2, 1 and 0) at position $\lambda$ with the SNP effect $\gamma$ at this same position, we obtain $\mathbf{Z}(\lambda)f(\lambda)=\widehat{VGA}$. Since $\widehat{VGA}+\varepsilon=y$, we have $y=\mathbf{Z}(\lambda)f(\lambda)+\varepsilon$which defines a functional model with a scalar response [20]. It is evident that the matrix product $\mathbf{Z}(\lambda)\gamma=\widehat{VGA}$represents the discrete sum of SNPs effects across the genome $\mathbf{Z}(\lambda)\gamma=\sum_{j=1}^{p} z_{(\lambda_{j})}\hat{\gamma}_{(\lambda_{j})}$; if so, their continuous counterpart can be using the following equivalence: $\mu+\mathbf{Z}(\lambda)f(\lambda)+\varepsilon\equiv\mu+\int_{0}^{L} \mathbf{Z}(\lambda)\gamma(\lambda)d\lambda+\mathbf{\varepsilon}=\mathbf{y}$ in which was described by Xu [9] as continuous genome where $\varepsilon$is the environmental error.

Therefore, if *y_i_* is the scalar phenotypic value of individual *i,* for *i = 1, ..., n,* the functional model that is adopted for *y_i_* considering one chromosome is:

$y_{i}=\mu+\int_{0}^{L} Z_{i}(\lambda)\gamma(\lambda)d\lambda+\varepsilon_{i}$ (1)

where *λ* is the marker position on the chromosome, which is expressed as a continuous quantity; *L* is the chromosome size; μ is the overall average; $\gamma(\lambda)$ is the marker response at the position *λ* (expressed as an unknown function of *λ*); **ε*_i_*** is the error related to individual *i* with$N\left(0,\sigma_{e}^{2}\right)$; and $Z_{i}(\lambda)$ is the state of the marker genotype at position *λ* for individual *i*, which is defined as:

$$\mathbf{Z}_{i}(\lambda)=\left\{\begin{matrix} 2 \text{for} AA \\ 1 \text{for Aa} \\ 0 \text{for aa} \end{matrix}\right.$$

The observed space of positions ($\lambda$) is discrete, but under saturation conditions it can be described by a continuous function. However, since not all positions $\lambda$ in the genome are known and there are many markers (with their respective positions), the positions are recorded in the original discrete space, where each sampled position ($\lambda\equiv M_{\lambda}$) has a corresponding signal $\gamma(\lambda)$.

Under high linkage disequilibrium among markers in a genomic window, $p(\lambda|\gamma,y)$ can be considered constant since $\mathbf{Z}(\lambda)$ is constant, by which one can be justify the models that were proposed by Hu et al. [10] and Xu [9,12], in which each artificial bin corresponds to a natural one. In addition, if *y* it not informative about $\gamma$, the posterior distribution $p(\lambda|y,\gamma)$ converges to a discrete uniform distribution, i.e., this artificial bin it not causal. In this respect, the conditional probability $p(\lambda|\gamma,y)$ can be integrated numerically through MCMC using the Metropolis-Hastings method.

Given a candidate value that is sampled within the *k-*th bin, a pseudo-functional model might be considered throughout the Metropolis-Hastings MCMC, which is reduced to a low-dimensional linear model:

$y_{i}=\mu+\sum_{m=1}^{K} Z_{im}\gamma_{m}+\varepsilon_{i}\text{,}$ (4)

where *K* is the number of bins in the current model and *m* refers to the *m-*th candidate marker in the *k*-th bin, which is stochastically sampled within the range $\left[\lambda_{k(\min)},\lambda_{k(\max)}\right]$.
