# Supplementary figures and images for: Functional models in genome-wide selection
Source: PLoS One. 2019 Oct 23;14(10):e0222699. doi: 10.1371/journal.pone.0222699 (PMC6808424; doi:10.1371/journal.pone.0222699)

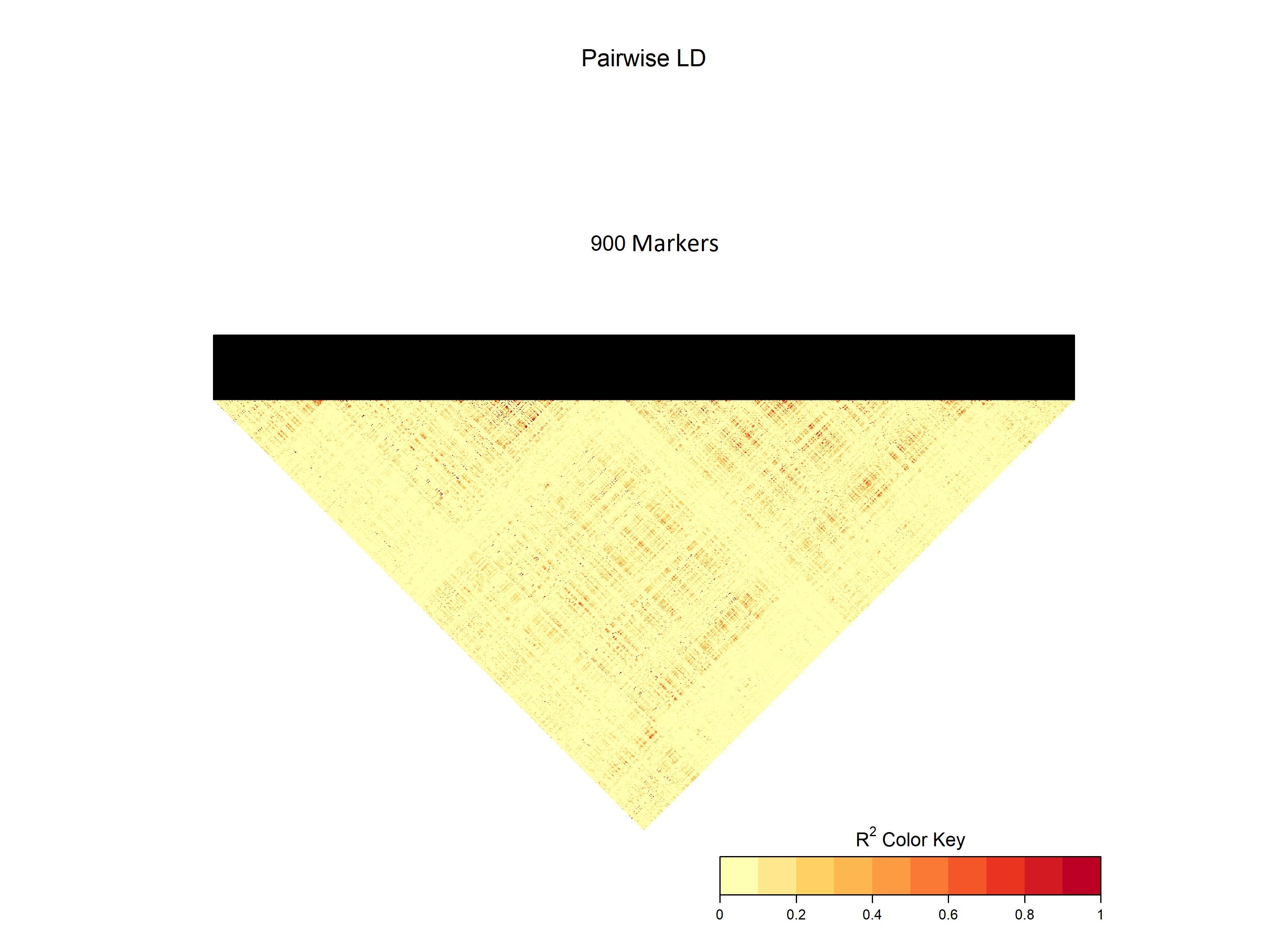

Supplement: S1 Fig — (JPEG) [file pone.0222699.s004.jpeg]

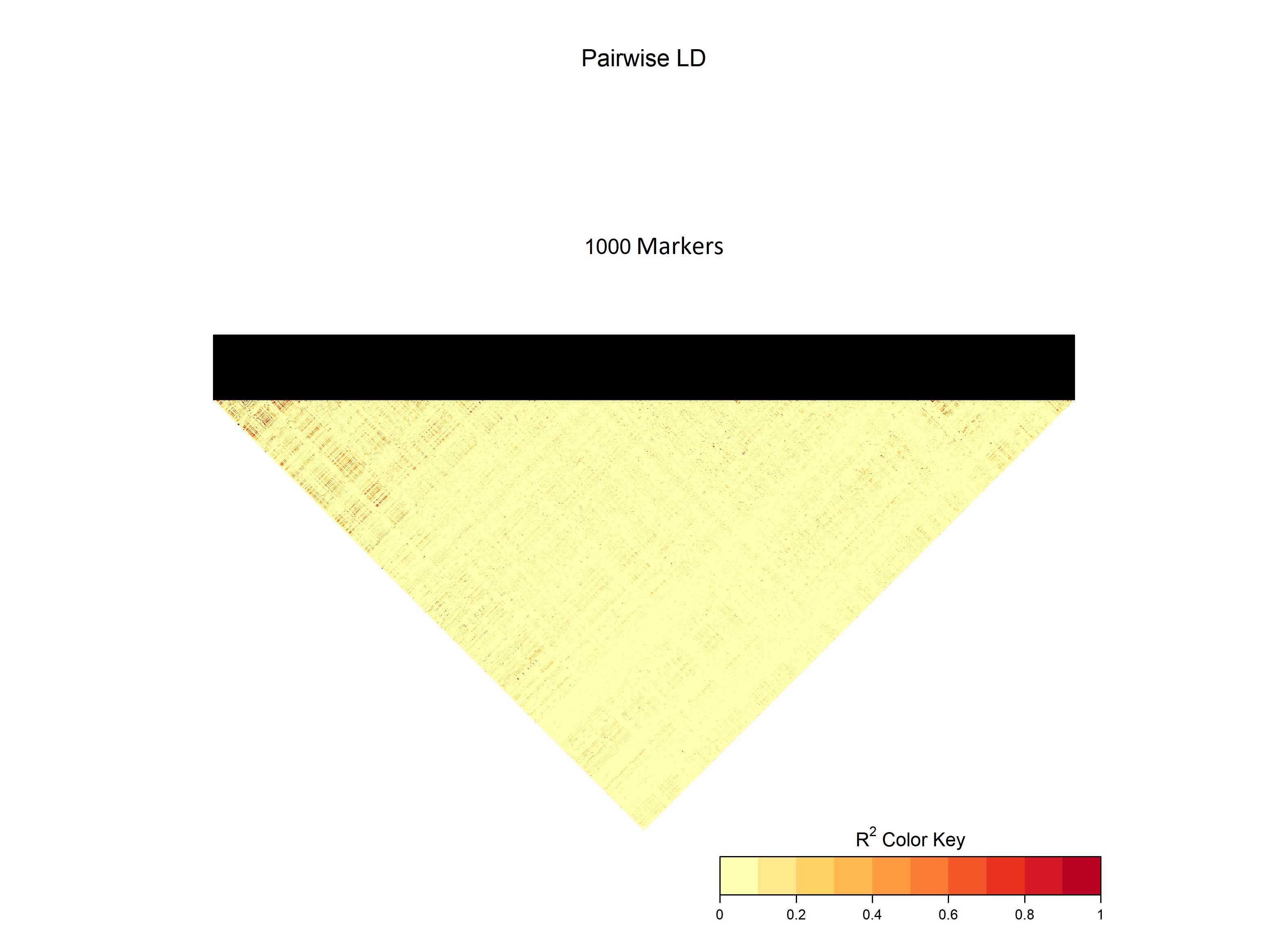

Supplement: S2 Fig — (JPEG) [file pone.0222699.s005.jpeg]

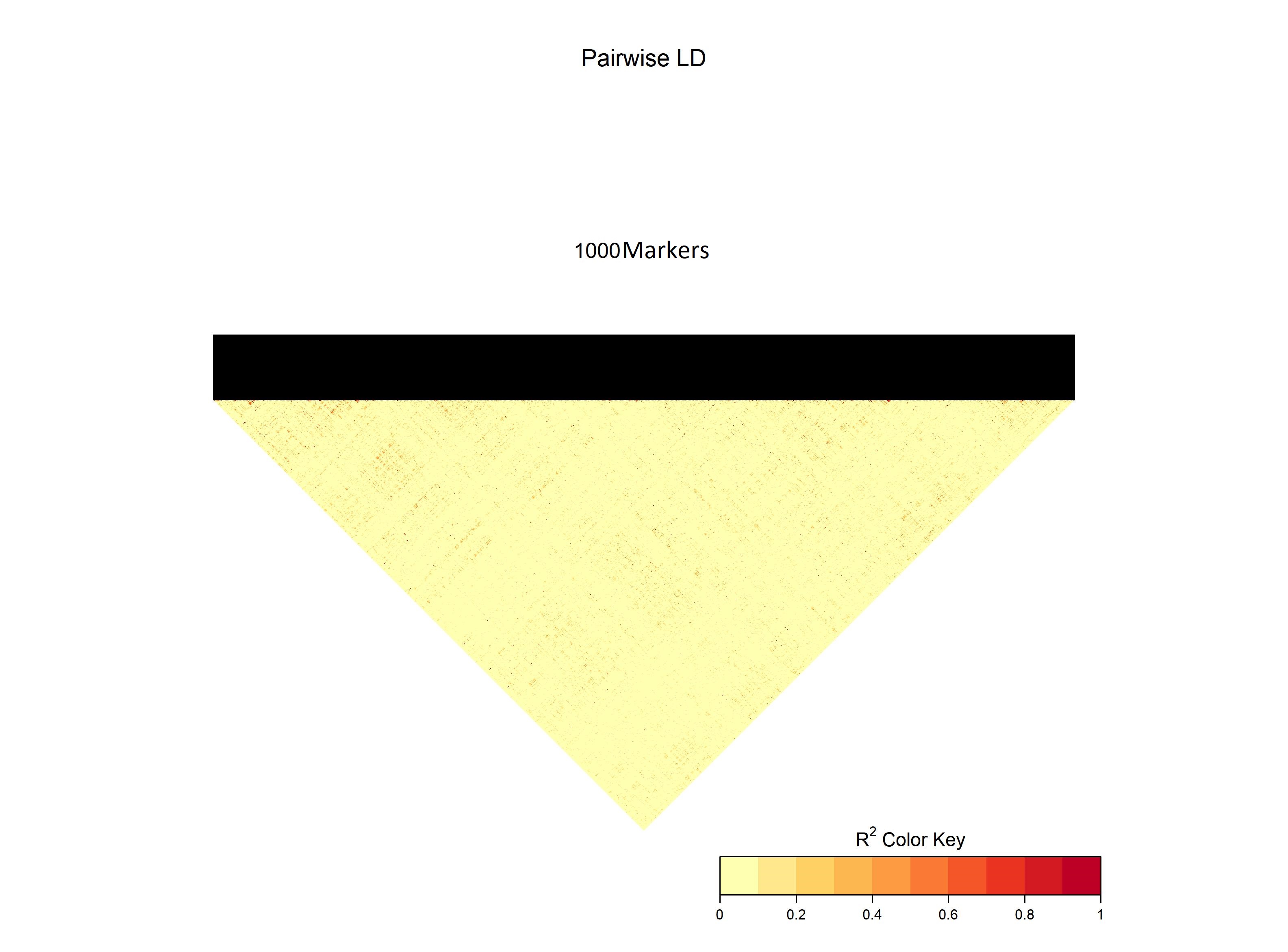

Supplement: S3 Fig — (JPEG) [file pone.0222699.s006.jpeg]

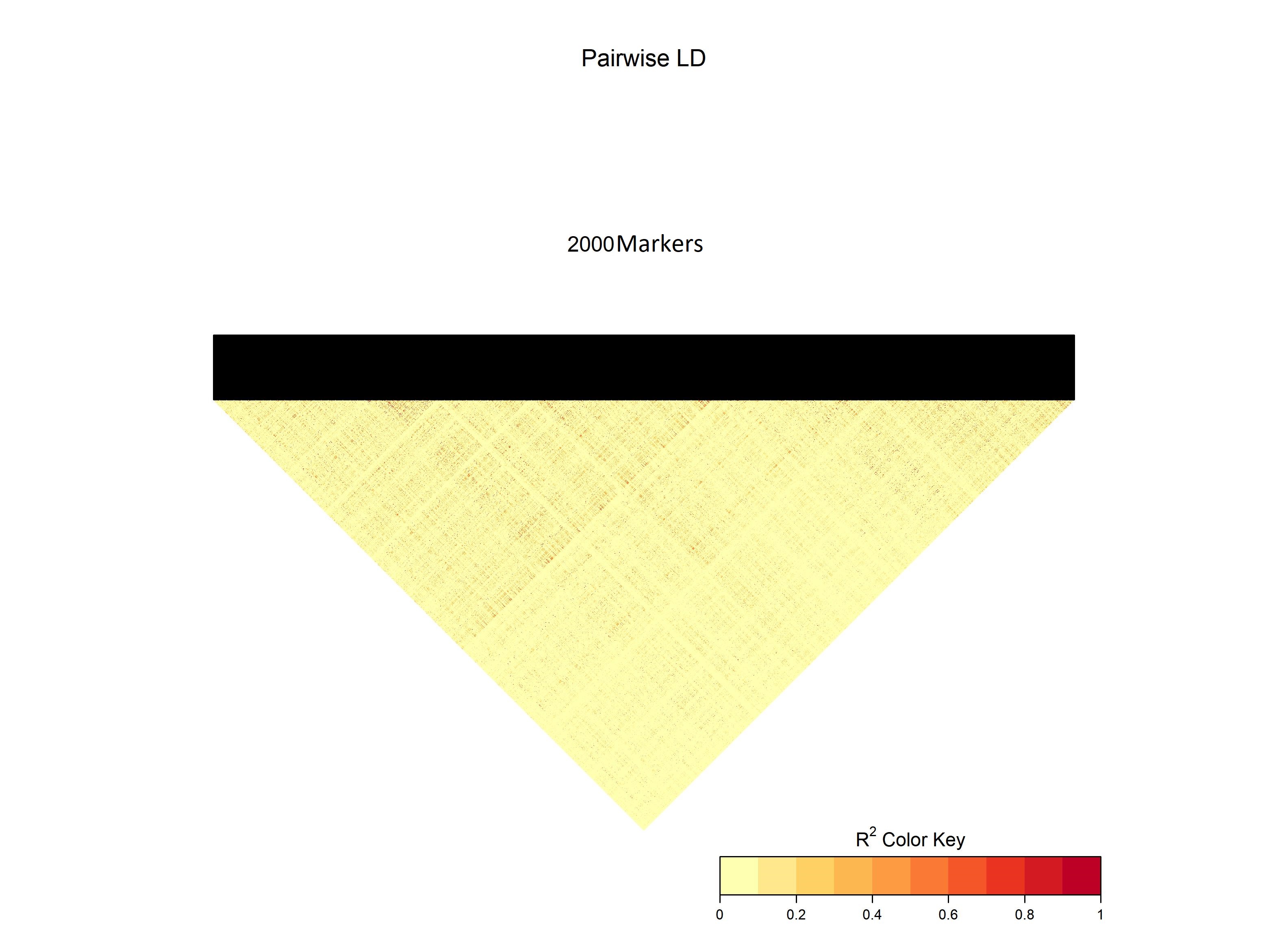

Supplement: S4 Fig — (JPEG) [file pone.0222699.s007.jpeg]

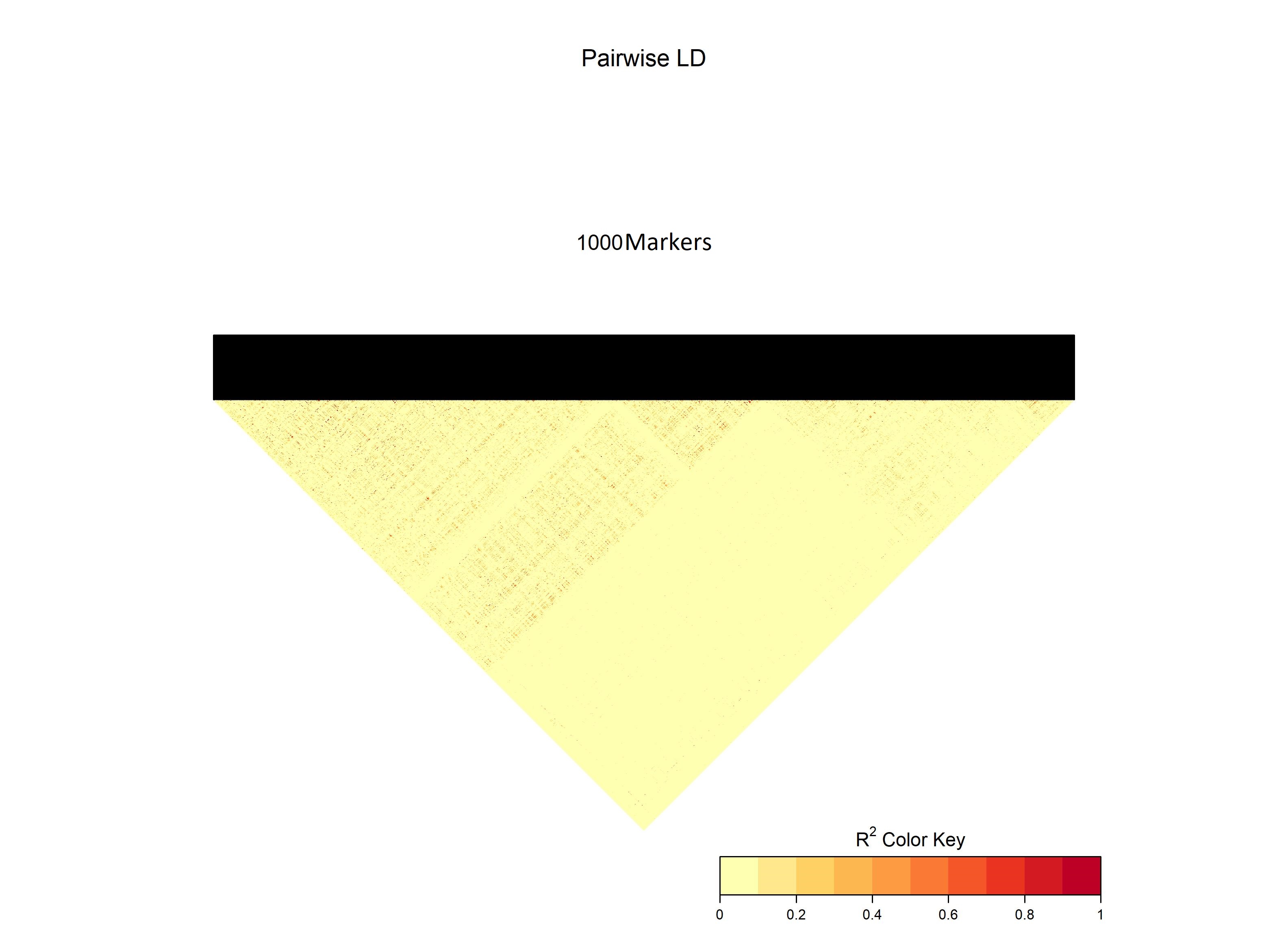

Supplement: S5 Fig — (JPEG) [file pone.0222699.s008.jpeg]

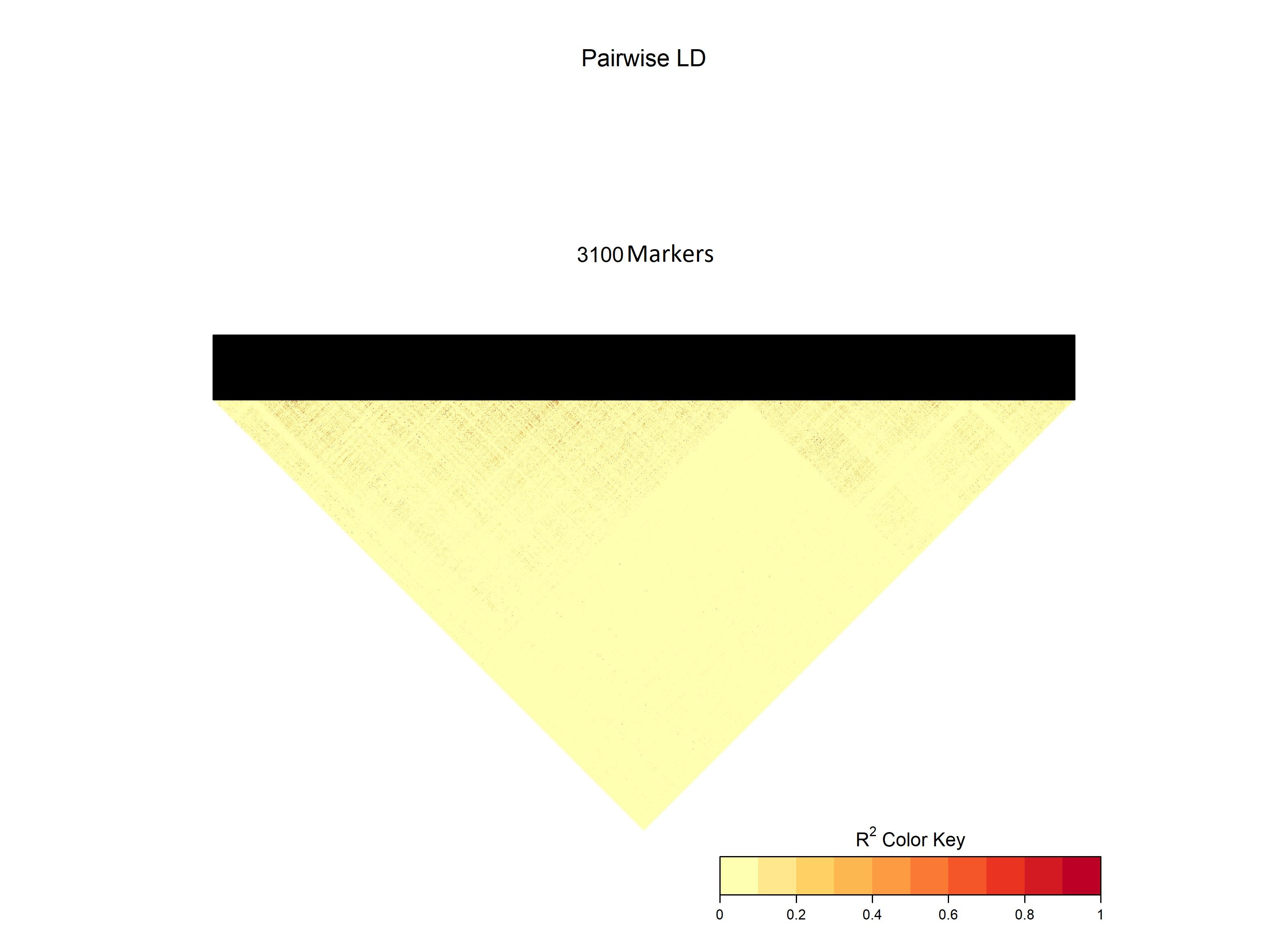

Supplement: S6 Fig — (JPEG) [file pone.0222699.s009.jpeg]

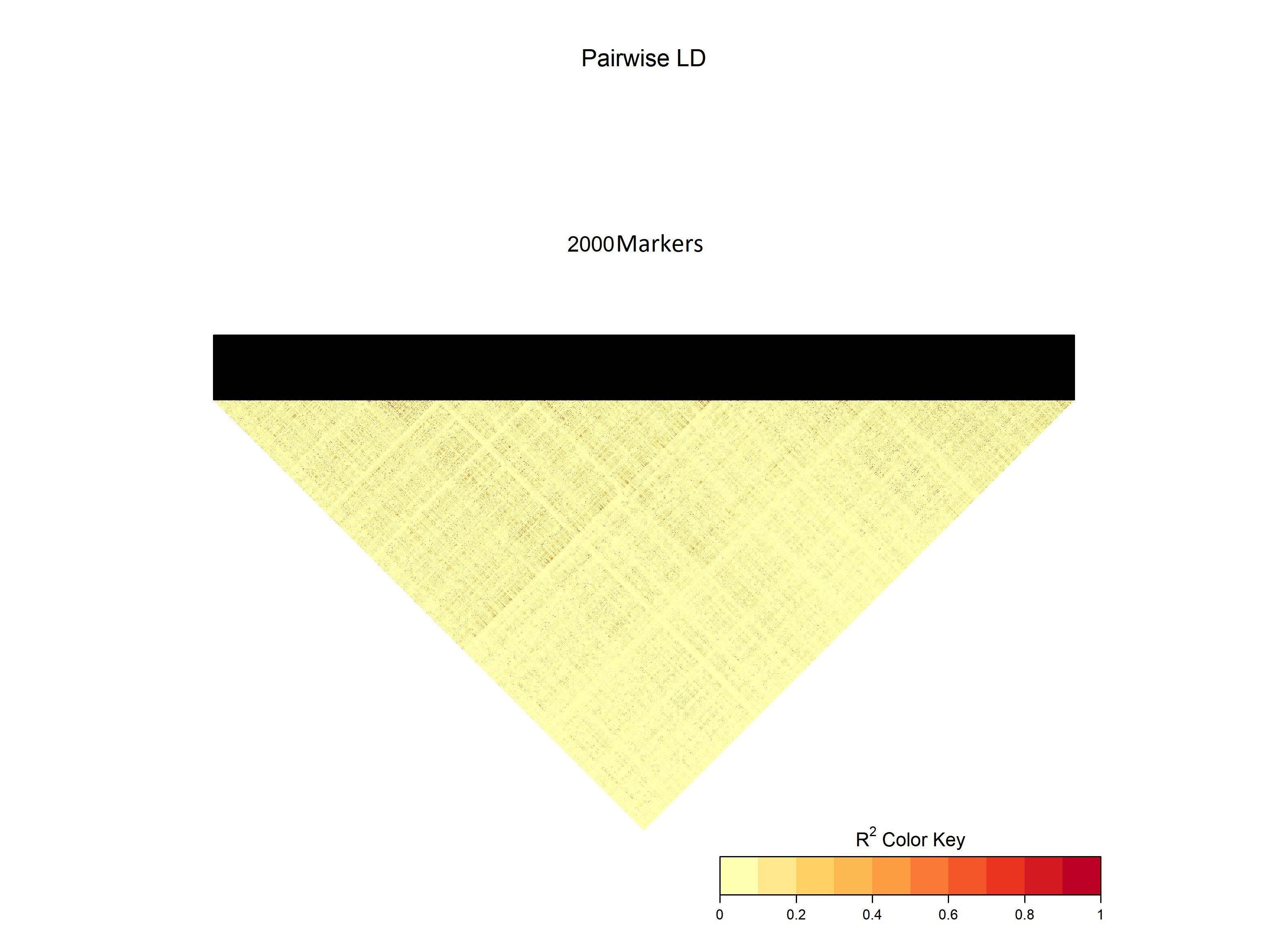

Supplement: S7 Fig — (JPEG) [file pone.0222699.s010.jpeg]

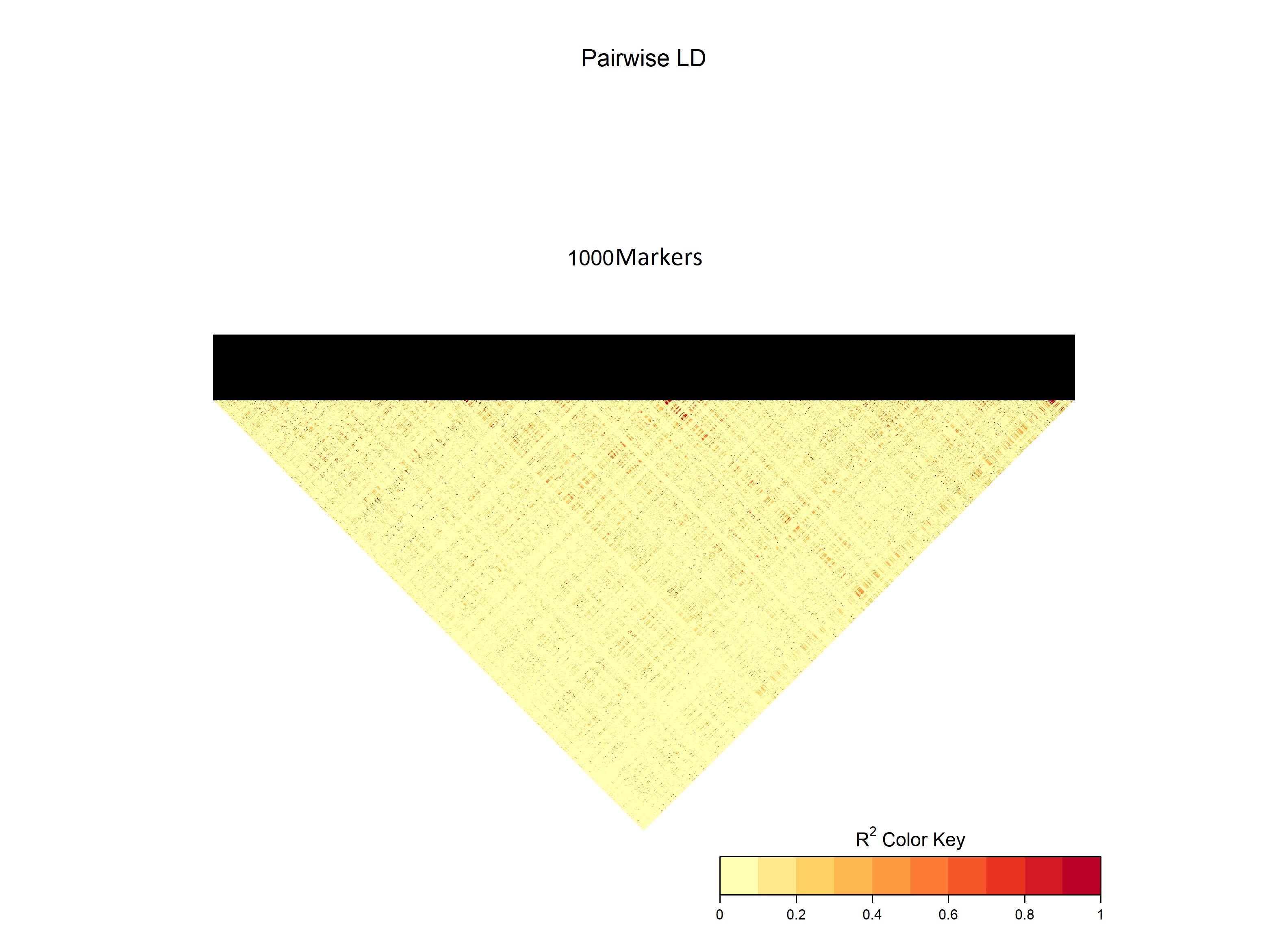

Supplement: S8 Fig — (JPEG) [file pone.0222699.s011.jpeg]

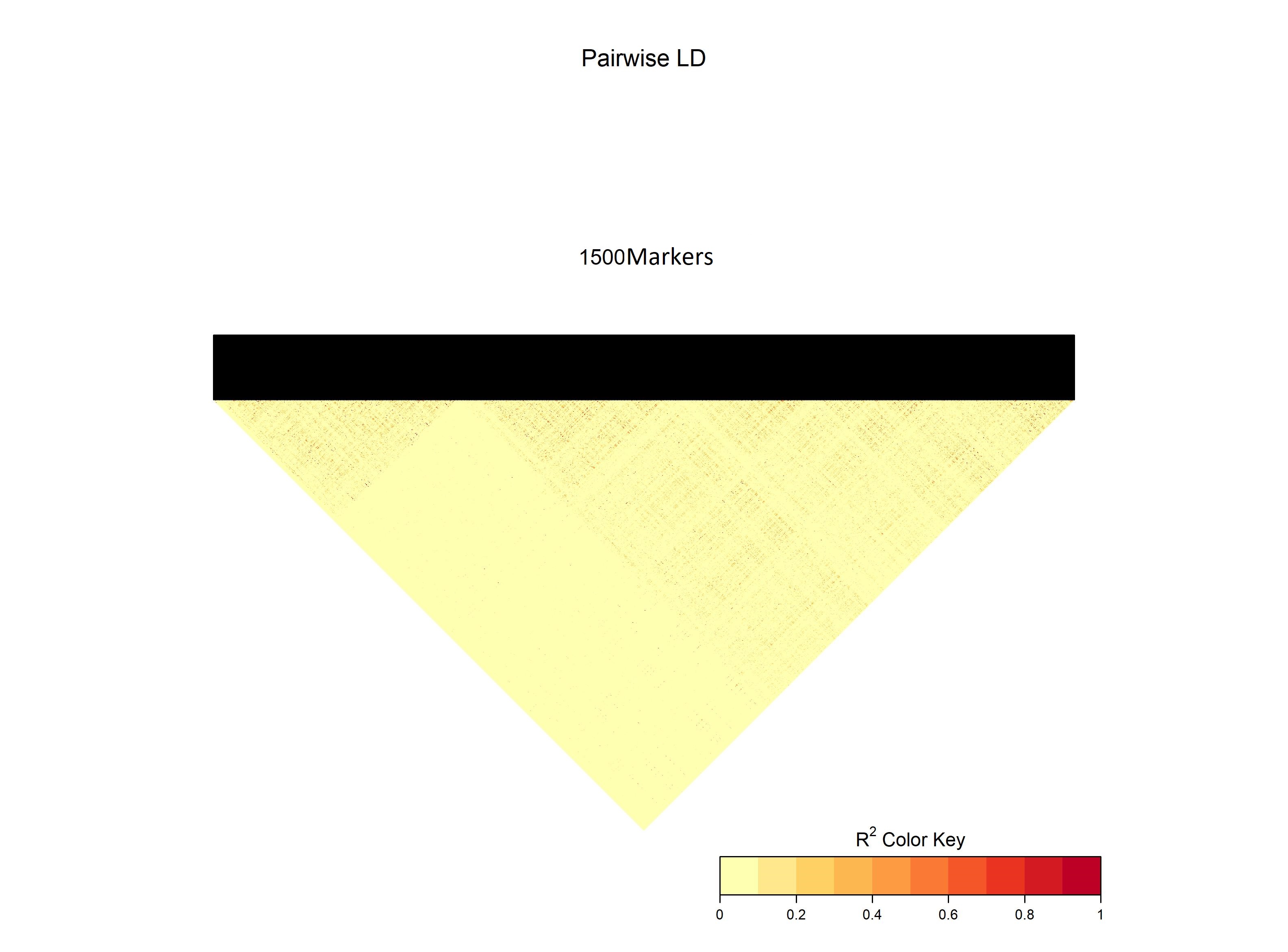

Supplement: S9 Fig — (JPEG) [file pone.0222699.s012.jpeg]

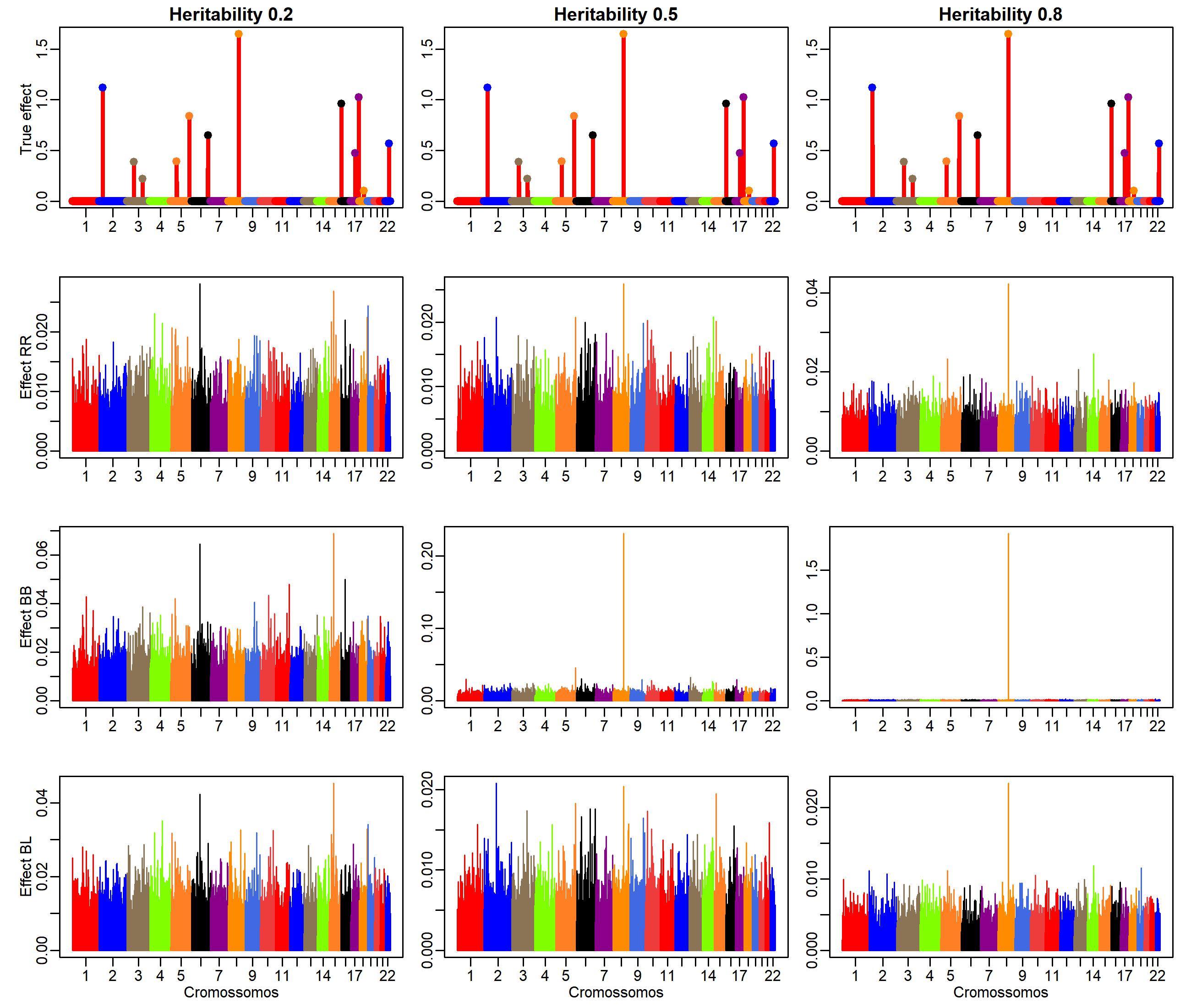

Supplement: S10 Fig — (JPG) [file pone.0222699.s013.jpg]

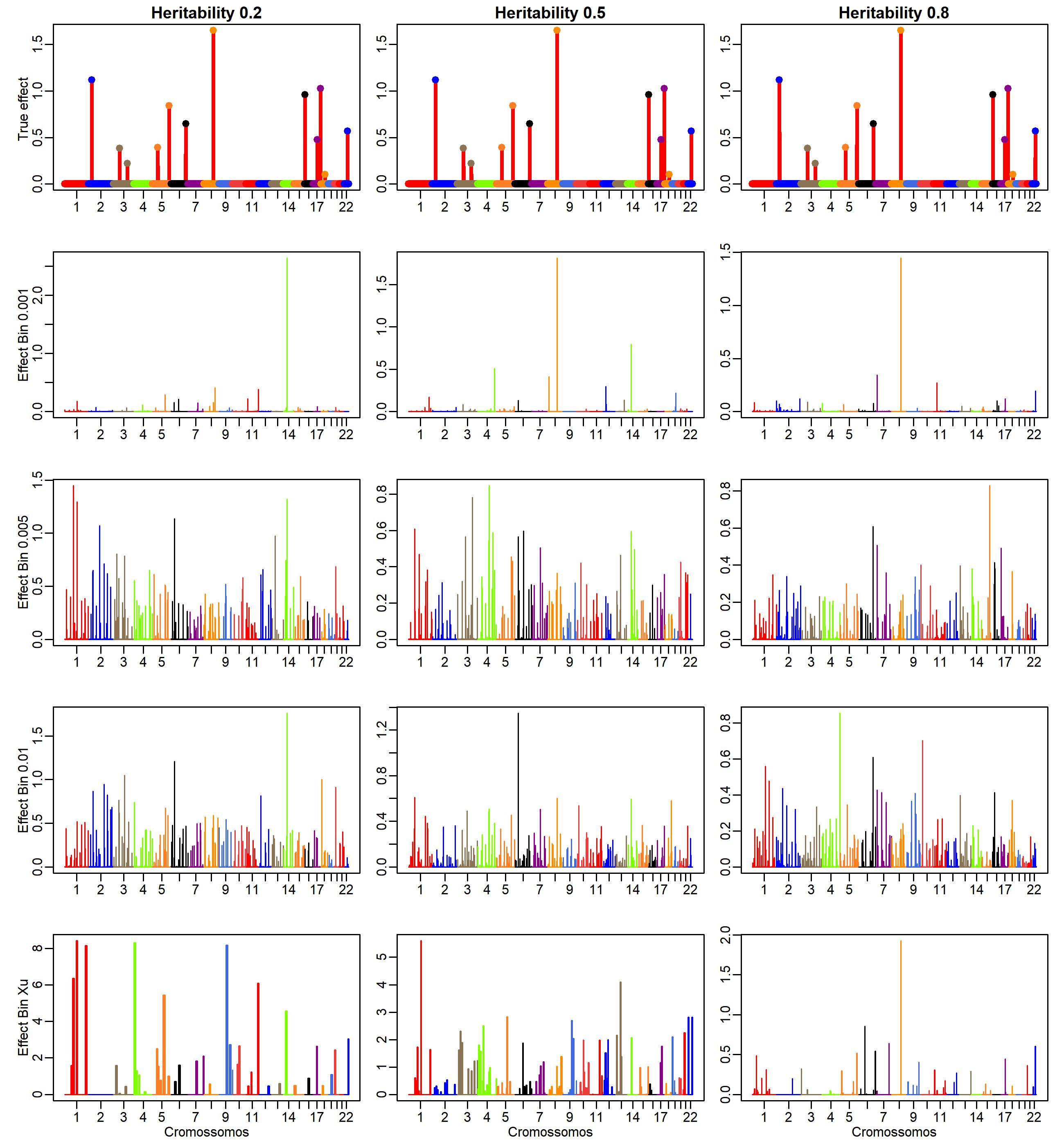

Supplement: S11 Fig — (JPG) [file pone.0222699.s014.jpg]
